# Towards precision oncology: unsupervised manifold learning for spatial molecular profiling in cancer tissues

**DOI:** 10.1186/s12859-026-06462-8

**Published:** 2026-05-15

**Authors:** Guoqing Jiang, Jingming He, Xuemeng Fan, Xiaoya Gao, Ran Huang, Cong Wu, Bairong Shen

**Affiliations:** 1 Department of Respiratory and Critical Care Medicine and Institutes for Systems Genetics, Frontiers Science Center for Disease-related Molecular Network, West China Hospital, Sichuan University, Chengdu, China; 2 Key Laboratory of Bio-Resource and Eco-Environment of Ministry of Education, College of Life Sciences, Sichuan University, Chengdu, China

**Keywords:** Nonlinear feature extraction, Precision oncology, Unsupervised learning, Manifold learning

## Abstract

Precision oncology relies on the accurate characterization of spatial molecular distributions in cancer tissues to uncover critical biomarkers and guide clinical decision-making. However, the high dimensionality and complexity of mass spectrometry imaging (MSI) data pose significant challenges for effective analysis. This study presents an unsupervised manifold learning framework to address these challenges by mapping high-dimensional MSI data into a low-dimensional space while preserving essential molecular patterns. This method enables efficient dimensionality reduction, clustering, and visualization of MSI data, facilitating the discovery of spatially resolved molecular features. Applied to datasets from prostate cancer and colorectal adenocarcinoma, the proposed method accurately identifies cancerous regions and reveals highly correlated molecular markers with Pearson correlation coefficients up to 0.79. These findings demonstrate the potential of unsupervised manifold learning to enhance the interpretability and utility of MSI data in precision oncology, paving the way for improved biomarker discovery and cancer diagnostics.

**Supplementary Information:**

The online version contains supplementary material available at 10.1186/s12859-026-06462-8.

## Introduction

Mass spectrometry imaging (MSI) is an analytical technique that effectively portrays the spatial distribution of molecules by combining mass spectrometry analysis with spatial imaging. This approach allows for the direct visualization of molecular distributions in solid samples or tissue sections.

Mass spectrometry imaging data analysis poses various challenges. Datasets are typically large, particularly with high-resolution, high-sensitivity instruments, which makes manual preprocessing a bottleneck in the overall analysis. Moreover, mass spectrometry imaging data, characterized by its high dimensionality, usually encompasses thousands of mass-to-charge ratio (*m/z*) channels, which leads to sparse data distribution in high-dimensional space and data complexity. This phenomenon is known as the "Curse of dimensionality" problem. Consequently, conventional machine learning methods struggle to support visualization and clustering of MSI data.

As the common dimensionality reduction methods, principal component analysis (PCA) and non-negative matrix factorization (NMF) are usually used for MSI data analysis [1, 2]. However, they can only present the linear mapping relations, which, in turn, hinders the capturing of complex nonlinear manifolds within the spectra.

In contrast, t-distributed stochastic neighbor embedding (t-SNE [3–7]) and uniform manifold approximation and projection (UMAP [8–12]), as nonlinear dimensionality reduction methods, become increasingly favored in omics data analysis over the past years [13, 14]. Nevertheless, t-SNE cannot project unseen data into the computed embedding. Additionally, the embedding results in t-SNE may undergo significant changes due to minor variations in the original data distribution. When it comes to UMAP, there are certain parameters that need to be adjusted, such as the number of neighbors and the minimum distance of points. In practical application, it is necessary to experiment with various combinations of these parameters to obtain the best result, which may introduce subjectivity. All of these problems may adversely affect the versatility and stability of the analysis.

Another unsupervised method, self-organizing maps (SOMs) [15, 16] can cluster sample regions containing benign cells and those containing malignant cells into distinct classes. However, due to the multiple iterations on the entire dataset, it can be time-consuming to train SOMs. Furthermore, the boundaries of true clusters in high-dimensional space may not be clear or may overlap, which makes the clusters derived from SOMs do not fully correspond to the true clusters of the original data.

**Fig. 1:**
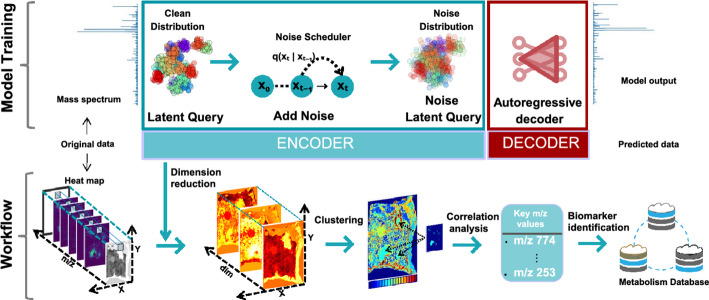
Training process and workflow of Atnal. (1) During training, Atnal undergoes iterative unsupervised learning, where the model’s loss is determined by comparing the original data with the predicted output. (2). In workflow, the raw data is dimensionally reduced using the trained encoder, and the reduced data is subsequently employed for clustering and correlation analysis. The decoder, however, is not utilized further. (3). Moreover, the ’Heat map’ and ’mass spectra’ represent the same dataset, differing only in their visualization methods

With the development of deep learning, the encoder-decoder architecture emerges as a promising tool in the field of bioinformatics. By designing appropriate encoder layers, it is possible to achieve efficient data compression while minimizing the loss of essential information. Based on this architecture, msiPL [17] is constructed by using variational autoencoders (VAEs). However, the backpropagation-based threshold analysis of msiPL may mistakenly exclude some crucial biomarkers.

In this context, we propose a novel neural network architecture called Atnal, to extend the unsupervised encoder-decoder framework. As the kernel of Atnal, the attention mechanism is widely applied in other fields, such as medical image processing [18], natural language processing [19], conventional computer vision [20, 21], and shows impressive capabilities. The incorporation of a noise diffusion model allows Atnal to effectively manage the inherent noise present in MSI data, enhancing the robustness of feature extraction. Additionally, this approach facilitates the preservation of critical molecular patterns during the encoding process, which is often compromised in traditional methods.

Key contributions are as follows:

i. An attention-based autoencoder model (Atnal) is introduced. To the best of current knowledge, it is the first unsupervised learning framework to integrate diffusion processes and autoregressive mechanisms for encoding raw MSI data.
ii. Atnal achieves computationally efficient, non-linear feature extraction that automatically captures latent molecular patterns, thereby ensuring a high-throughput workflow that is independent of sample properties and hardware configurations.
iii. Experimental results demonstrate that Atnal is superior to the baseline methods in terms of reconstruction error, thereby validating the effectiveness of our approach in metabolomic data analysis.

**Fig. 2:**
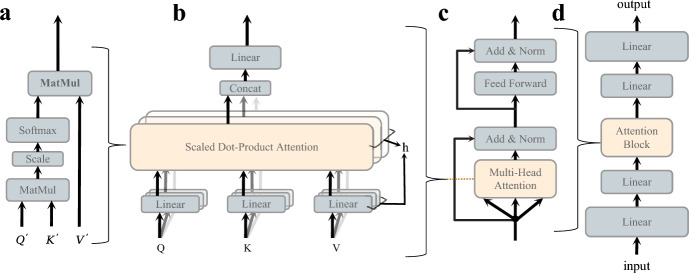
Detailed network architecture of Atnal

## Methodology

### Diffusion-based encoder with autoregressive modeling

Atnal utilizes a diffusion-based encoder to effectively manage and interpret spectral data from mass spectrometry. The encoder starts with a latent representation of the original mass spectrum and subsequently applies a controlled noise injection process. This process employs a forward diffusion model that iteratively adds Gaussian noise to the latent representation, thereby facilitating a gradual corruption of the clean signal.

To optimize the noise addition, a noise scheduler is implemented, which governs the amount of noise introduced at each diffusion step. This allows Atnal to contextualize important spectral features while decreasing the influence of random noise. This method not only enhances the robustness of the encoder but also aids in extracting meaningful patterns from the inherently noisy data typical of mass spectrometry.

Atnal’s encoder leverages an autoregressive model, which sequentially processes the noisy inputs derived from the diffusion steps. This structure enables the model to utilize previously learned latent variables to predict the next element in the sequence, effectively employing a learned prior distribution for noise reduction. By doing so, the autoregressive mechanism enriches the encoding process, ensuring that noise is mitigated over successive iterations.

### Experimental datasets

The study uses publicly available MSI datasets for two distinct tissue types: human colorectal adenocarcinoma and human prostate cancer. The datasets consist of a 3D DESI MSI dataset for colorectal adenocarcinoma [22] and a 2D MALDI FT-ICR MSI dataset for prostate cancer [17].

The colorectal adenocarcinoma dataset, in the mass range* m/z* 200–1050, from a single colorectal adenocarcinoma specimen (26 sections), is acquired under the negative-ion mode. This dataset contains the metadata, coregistered imaging MS data, and optical [haematoxylin and eosin (H&E)-stained] images. It can be accessed at http://gigadb.org/dataset/100131. Within this dataset, the spatial resolution during acquisition is set to 100 $\mu$m. The dataset possesses 148044 spectra, of which each contains 8073 dimensions.

As for the prostate cancer dataset, it is acquired from a 9.4 Tesla SolariX XR FT ICR mass spectrometer (Bruker Daltonics, Billerica, MA) using the MALDI source in positive ion mode. The range of* m/z* is from 250 to 1000, and the spatial resolution is 120 $\mu$m. Internal online calibration is performed using heme* m/z* 616.1776 during data acquisition. This dataset is now available at the National Metabolomics Data Repository (NMDR) Metabolomics Workbench www.metabolomicsworkbench.org under project id (PR001171) with https://doi.org/10.21228/M8BM4Q.

### Data processing

There are four vectors in each dataset: $Crd_x$, $Crd_y$, *X*, and *MzList*.

As a one-dimensional vector, the *MzList* stores different* m/z* values, and its length is defined by *d*. $$ X = \{ x^{(1)},x^{(2)},...,x^{(n)}\}$$ represents relative abundance of ions detected by MSI instrument where *n* represents the overall length of *X* and the count of sampling points. Moreover, each spectrum $$x^{(i)} \in \mathbb {R} ^{d}$$ is of *d*-dimensions. As for $$ Crd_x$$ and $$Crd_y$$, they are both one-dimensional vectors with a length of *n*, and they contain spatial location information corresponding to each point.

Prior to preprocessing, spectra with all intensities of 0 are found in the colorectal adenocarcinoma dataset; these zero-intensity spectra are removed.

In order to mitigate the negative impact of the significant abundance differences between sampling points on the subsequent encoding performance of the model that will be built later, the vector *X* is normalized by using the so-called total-ion-count (TIC) normalization. Specifically, the normalized *X* can be given as:

1 $$\begin{aligned} X_{i,j}= \frac{X_{i,j}}{\sum _{k=1}^{d}X_{i,k}} \end{aligned}$$

where $$i\in [1,n]$$.

### Training an attention-based autoencoder model

In this study, the Attention-Based autoencoder model (Atnal) is used for extracting the nonlinear features of MSI data. It is performed using Python (v.3.8.16), Pytorch (v.1.13.1) and CUDA (v.11.7.1), specifically, Atnal is trained on a Quadro P4000 GPU. In training, an Adam optimizer with an initial learning rate of 0.0001 and an early stopping strategy are employed by monitoring the model loss in the testing set. If the loss of the testing set does not decrease in 4 epochs, the model training stops and the best model is recorded. Moreover, the input of Atnal, a set of the dimension $n \times d$, includes an ultrahigh spectral resolution of 2D FT-ICR MSI of prostate cancer tissue (all for training) and a 3D DESI MSI dataset of a human specimen of colorectal carcinoma (80% of 26 consecutive sections for training and 20% for testing).

Architecture of Atnal: Atnal adopts an encoder-decoder architecture, to facilitate efficient unsupervised learning and nonlinear dimensionality reduction by using neural networks. The encoder, composed of a Multilayer Perceptron (MLP) and an Attention Block [23] (Fig. 2d), maps an input sequence of symbol representations $X=( x^{(1)},...,x^{(n)})$ to a sequence of continuous representations $Z=(z^{(1)},...,z^{(n)})$. It is used as a recognition model to infer an approximate estimate of the true but intractable distribution of the latent variable *z* underlying the complex high-dimensional MSI data *X*. The decoder, a MLP, generates an output sequence $Y=(y^{(1)},...,y^{(n)})$ from *Z*. The decoder is used as a generative model to reconstruct the data by solely utilizing the encoded features *Z*. Both the parameters of the recognition model (encoder) and the generative model (decoder) are jointly optimized via backpropagation. Thus, the data flow of Atnal is as follows:

2 $$\begin{aligned} encoder(X) = Attention \, Block(MLP_e(X)) Atnal(X)=MLP_d(encoder(X)) \end{aligned}$$

where $X=( x^{(1)},..., x^{(n)})$. $Attention \, Block$ will be introduced later. $MLP_e$ and $MLP_d$ are two different Multilayer Perceptrons mentioned in the encoder and decoder, respectively.

Attention Block: Here we introduce the Attention Block in detail, starting from the fundamental module: Scaled Dot-Product Attention (Fig. 2a) [23]. Its input consists of queries ($Q'$) and keys ($K'$) of dimension $n \times d_k$, and values ($V'$) of dimension $n \times d_{v}$. The dot products of the queries are calculated with all keys, then divided by $\sqrt{d_{k}}$. Finally, a softmax function is applied to obtain the weights of the values. Consequently, the output of Scaled Dot-Product Attention can be given by

3 $$\begin{aligned} Attention(Q',K',V')=Softmax(\frac{Q'K'^T}{\sqrt{d_{k}}})V'. \end{aligned}$$

The Scaled Dot-Product Attention is the kernel of Multi-Head Attention, as illustrated in Fig. 2b. Using queries (*Q*), keys (*K*) and values (*V*) with dimension of $n \times d_{model}$ (different from $Q'$,$K'$,$V'$ mentioned earlier) as inputs, the Multi-Head Attention allows Atnal to jointly attend to information from different representation subspaces at different positions. The queries, keys and values are projected *h* times by distinct, learnable linear projections to $d_k$, $d_k$ and $d_v$ dimensions, respectively. As a result, the data flow of Multi-Head Attention can be expressed as follows:

4 $$\begin{aligned} MultiHead(Q,K,V)= Concat(head_1,head_2,...,head_h)W^O \end{aligned}$$

where $$W_i^O \in \mathbb {R}^{hd_v \times d_{model}}$$ and

5 $$\begin{aligned} head_i = Attention(QW_i^Q,KW_i^K,VW_i^V) \,\, \end{aligned}$$

where the projections are learnable parameter matrices $W_i^Q \in \mathbb {R}^{d_{model} \times d_k}$, $W_i^K \in \mathbb {R}^{d_{model} \times d_k}$ and $W_i^V \in \mathbb {R}^{d_{model} \times d_v}$. Throughout this paper, we employ h = 8 parallel attention heads. Moreover, we select $d_k$ = $d_v$ = $d_{model}/h$ = 32 for each of these heads.

Finally, the Attention Block is structured by using two sub-layers. The first sub-layer consists of a multi-head attention mechanism, while the second sub-layer is a simple, fully connected feed-forward network (FFN). To ensure smooth information flow, a residual connection [24] is adopted around each of the two sub-layers, followed by layer normalization [25]. In general, the data flow from the input of the Attention Block to its output is as follows:

6 $$\begin{aligned} Q,K,V&= input \cdot \begin{bmatrix} W^1,W^2,W^3 \end{bmatrix} \end{aligned}$$

7 $$\begin{aligned} output_{mul}&= LayerNorm( Residual([Q,K,V], MultiHead)) \end{aligned}$$

8 $$\begin{aligned} output&= LayerNorm({Residual}( output_{mul}, FFN)) \end{aligned}$$

where *input* is the TIC-normalized spectra mentioned in Data Processing, and $W^1,W^2,W^3 \in \mathbb {R}^{d \times d_{model}}$ are learnable parameter matrices of three linear neural networks which aim to project *input* into three matrices *Q*, *K*, *V*. Besides, the function $\textrm{Residual}(X,\text {sublayer})$ is:

9 $$\begin{aligned} Residual(X,\text {sublayer}) = X + sublayer(X). \end{aligned}$$

Loss function: Since the reconstructed MSI data $Y = ( y^{(1)},...,y^{(n)})$ is a sparse matrix, the dissimilarity between *Y* and the original data $X=( x^{(1)},...,x^{(n)})$ can be computed as the error of a multi-classification problem. Here, the dissimilarity is derived from the loss function of Atnal, given by

10 $$\begin{aligned} \text {Loss} =\frac{1}{n} \sum _{i=1}^{n}H(X^{(i)}|Y^{(i)}) \end{aligned}$$

where *H* (categorical cross-entropy loss [26]) is

11 $$\begin{aligned} H(P^{*}|P) = -\sum _{i}^{}P^{*}(i)\log P(i) \end{aligned}$$

where $P^*$ and *P* represent the true class distribution ($X^{(i)}$) and predicted class distribution ($Y^{(i)}$), respectively.

### Correlation analysis and tentative metabolite annotation

The encoded features represent the learned nonlinear manifold in lower-dimensional space, which can promote the visualization and extraction of molecular patterns from original high-dimensional data. The inherent complexity of the original high-dimensional data is significantly reduced, which makes it possible to apply straightforward clustering algorithms such as the Gaussian mixture model (GMM) [27]. The GMM clustering results show that distinct clusters correspond to different regions on the tissue. For better performance of clustering, the users have the flexibility to determine the number of clusters (k) for the GMM algorithm. To determine the optimal number of clusters, the Bayesian Information Criterion (BIC) is utilized. To mitigate the inherent variability associated with stochastic initialization, BIC evaluations are repeated over 20 iterations using distinct random seeds by varying the random_state parameter in Python package: sklearn.mixture.GaussianMixture. The final cluster count is determined by the modal value across these iterations, a strategy designed to reduce the influence of random artifacts and ensure the statistical stability of the findings. Subsequently, a cluster of interest is selected for correlation analysis, which aims to determine a few colocalized* m/z* peaks associated with high Pearson correlation coefficients.

For tentative metabolite annotation, initially, the determined *m/z* values are input into comprehensive databases of human metabolites, including the Human Metabolome Database (HMDB) [28], the LIPID MAPS®Structure Database (LMSD [29]), and MetaboAnalyst Database [30]. For each *m/z* value, the matched compounds in the databases are considered as putative annotations only, not definitive metabolite identifications. A *m/z* match in the database indicates a preliminary hypothesis that requires additional validation through orthogonal methods (e.g., tandem MS/MS, co-elution with standards). Here, the tolerance window is set lower than 5 ppm. If none of the three aforementioned databases can be used to find a match for a given *m/z* value, then this *m/z* value will be represented only by its value, indicating that no known metabolite can be putatively assigned for this specific value.

## Results

### Hyperparameters and implementation details

The original MSI data is analyzed via Atnal, an encoder-decoder model based on attention mechanism and multi-layer perceptron (MLP), as depicted in Fig. 1.

The proposed neural network architecture is comprised of five layers: an *Input* layer ($L_1$), three hidden layers: $MLP_e (h_2)$, $Attention \, Block (h_3)$ and $MLP_d (h_4)$, and an *Output* layer ($L_5$). As shown in Table 1, the number of artificial neurons of $L_1$ or $L_5$ is the same as that of *m/z* bins, and the dimension of the output from hidden layers $h_2$, $h_3$, and $h_4$ is 256, 256 and *m/z* dimensions, respectively. The $Attention \, Block$ ($h_3$) contributes to capturing the encoded features that represent the non-linear dimensionality reduction of original MSI data. Layers $h_2$, $h_3$, and $h_4$ are followed by batch normalization. Further, the rectified linear unit [31] (ReLU) function is used for neuron activation in the output of hidden layers $h_2$ and $h_4$. As for the *Output* layer $L_5$, its neurons are activated by the softmax function.

In our model, the initial layers contain a larger number of neurons, facilitating the extraction of rich features from the input data. As the network deepens, the layers progressively narrow, aiding in the compression and aggregation of these features. This narrowing forms a "bottleneck" in the intermediate layers, which compels the network to compress information and extract the most significant features.

Many successful network architectures, such as U-Net [32], VGG [33], and ResNet [24], have employed this layer design strategy, which has proven effective in practice.

From a macro view in Atnal, the unsupervised learning process involves minimizing the reconstruction error between the original and predicted data. The minimization is realized through optimizing the cost function which is modeled as the marginal likelihood and calculated via categorical cross-entropy; The Adam stochastic gradient optimizer is employed with a learning rate of 0.0001, in which the training is performed on mini batches consisting of 512 spectra for a total of 20 epochs.

**Table 1:** Dimensions of the output of each layer

Input	MLP$_e$		Attention	MLP$_d$		Output
	Linear$_{e1}$	Linear$_{e2}$		Linear$_{d1}$	Linear$_{d2}$	
*m/z* dim	512	256	256	512	*m/z* dim	*m/z* dim

**Fig. 3:**
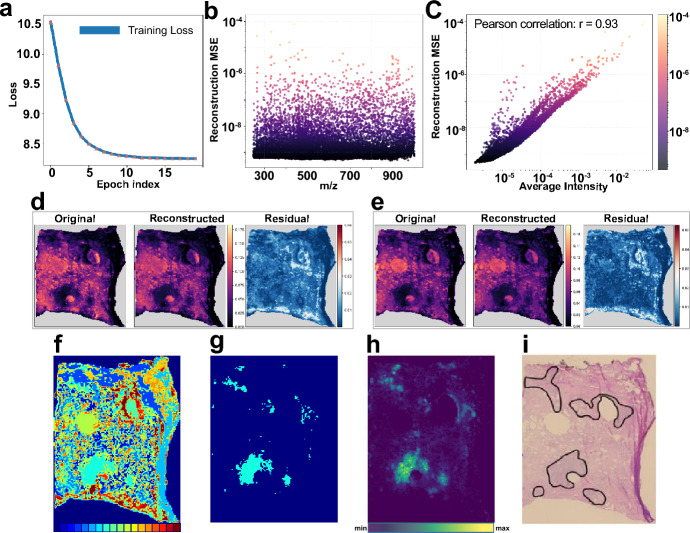
**a** Training loss curve spanning 20 epochs. **b** Mean Squared Error (MSE) averaged across all spectra. **c** MSE versus average ion intensity. **d**,** e** Visualization of the reconstruction error for the two most erroneous ions, 455.01 and 439.04. More visualizations of reconstruction results can be found in Supplementary Fig. 2. **f** Clustering outcomes of encoded features compressed by Atnal’s encoder, cluster number k = 17 + 1 (blank background cluster). **g** Chosen cluster associated with cancer. **h** Molecular pattern of* m/z* 774.5983, which exhibits the highest correlation with (**g**). **i** Histopathological annotation of tumor regions. H&E-stained tissue images obtained after MALDI MS imaging. Tumor regions identified by histopathological evaluation are marked in black. The Gleason score (GS) is (3 + 4) = 7 [34]

### Analysis of the 2D FT-ICR MSI data from prostate cancer tissue

Aiming to validate the efficacy of Atnal in encoding features and reconstructing original data, Atnal is applied to analyze the entire prostate cancer dataset. The results are presented in Fig. 3.

The neural network employs unsupervised learning in an iterative fashion to minimize the reconstruction loss. As depicted in Fig. 3a, the optimizer achieves convergence in fewer than 15 epochs, with a total running time of approximately 6 min. The encoded features, illustrated in Supplementary Fig. 5, serve as a nonlinear embedding that facilitates visualization and reveals molecular patterns within a compressed representation of the original high-dimensional data (shown in Supplementary Fig. 1). The generative model reconstructs the original data by only using these 256-dimensional encoded features. The reconstructed spectra achieve a mean squared error (MSE) of $7 \times 10^{-7}$ compared to normalized inputs. Fig. 3b illustrates the MSE averaged across all spectra. Furthermore, Fig. 3c displays the MSE versus average ion intensity, indicating that Atnal performs well across ions of varying intensities. 3d and e illustrate the reconstruction results of the two ions with the highest reconstruction errors, 455.01 and 439.04. These visualizations demonstrate Atnal’s capability for low-error reconstruction of spectra.

The histopathological annotation of the prostate tumor region reveals a Gleason score (GS) of (3 + 4) = 7 (Fig. 3i) [35]. The understanding of molecular patterns underlying the annotated histopathological tumor regions could contribute to the development of molecular diagnostics. The encoded features are clustered by the Gaussian-mixture model (GMM) with k clusters (k = 17) (Fig. 3f). Among these clusters, a certain one (Fig. 3g), presenting the cancerous regions that are consistent with the histological evaluation (H&E) in Fig. 3i, shows the highest correlation at* m/z* 774.5983 (Fig. 3h).

Among the 15* m/z* values with top-ranked correlation coefficients, 7 yield putative database annotations within a 5 ppm tolerance. For example, the* m/z* value 774.5983 indicates 16 potential phosphatidylcholine and phosphatidylethanolamine metabolites.

Abnormal phospholipid metabolism is a key indicator of tumors. These phosphatidylcholine and phosphatidylethanolamine metabolites, as the products of phospholipid metabolism, play crucial roles in maintaining cellular membrane integrity and regulating lipid-dependent signaling pathways. The functions of phosphatidylcholines and phosphatidylethanolamines in the prostate cancer are also reported in several studies [36–40].

During the compound matching, a few signal molecules related to fatty acid metabolism are also discovered, such as *m/z* 762.5862 and *m/z* 804.6077. Among them, the *m/z* 804.6077 is identified as 12 potential compounds which belong to the class of glycerophospholipids and previously identified in prostate cancer samples in the database: Metabolomics Workbench (https://www.metabolomicsworkbench.org), with study IDs ST000784 and ST001133. For *m/z* 762.5862, a neutral glycosphingolipid candidate is indicated (error 1.00 ppm), subject to confirmation. For more information on compound identification, please refer to Supplementary Table 1.

**Fig. 4:**
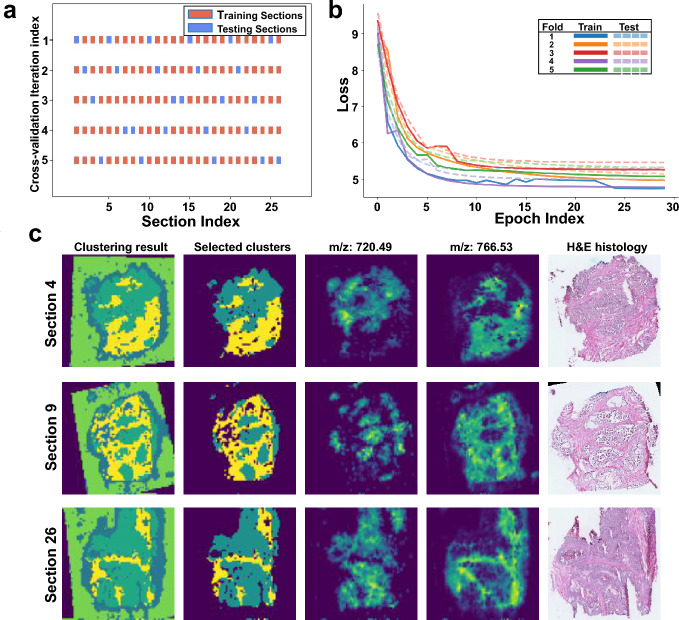
Analysis on colorectal carcinoma dataset (Training phase). **a** 5-fold cross-validation analysis: The full MSI dataset is randomly shuffled and split into an 80% training set and a 20% testing set, and this procedure is iterated five times. **b** Training and testing loss curves of the 5-fold cross-validation. **c** Encoded features (Supplementary Fig. 6) are clustered by GMM (k = 5; only 4 clusters are observed here; blank background yields a fifth color). Two clusters align with cancerous and connective tissue in H&E histology, respectively. Two ion channels show the highest correlation with the two clusters, respectively

**Fig. 5:**
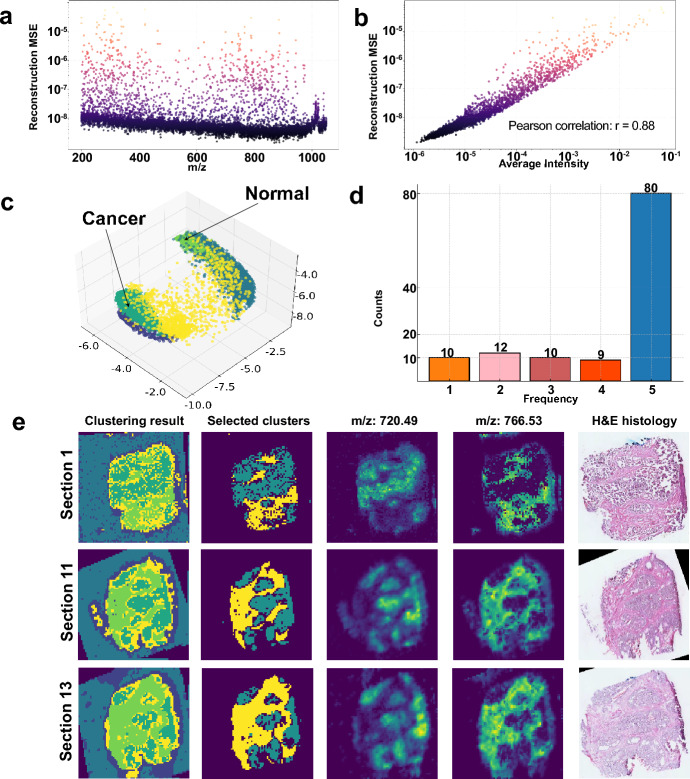
Analysis of colorectal carcinoma dataset (Testing phase). **a** Mean Squared Error (MSE) averaged across all spectra. **b** MSE versus average ion intensity. **c** The distribution of encoded features in a 3-dimensional space, which is reduced from 256 dimensions to a 3-dimensional space by using PCA for the purpose of visualization. The spectra that respond to cancerous tissue demonstrate spatial similarity, as do the spectra of normal (connective) tissue. These results in this phase are also derived from the moderate model as mentioned in the training phase. **d** The statistical analysis encompasses all top 100 correlated* m/z* values derived from the 5-fold cross-validation, yielding a total of 121 distinct values. The majority of these values are consistently present across all cross-validation iterations, demonstrating the robustness of the Atnal-derived ion list. (e): Those encoded features (Supplementary Fig. 7) are clustered by GMM (k = 5; the blank background shows up as a sixth color), and two clusters are selected, matching cancerous and connective tissue in H&E histology, respectively. Two ion channels show the highest correlation with the two clusters, respectively

### Analysis of the 3D DESI MSI data from colorectal adenocarcinoma dataset

Here, Atnal is used to reconstruct a 3D MSI volume from a human colorectal adenocarcinoma specimen: 26 consecutive (acquired at every 100 $\mu$m) 10 $\mu$m thickness tissue sections. To reduce the impact of imbalanced data splitting on model loss, we implement K-fold cross-validation during the process of the model training (Fig. 4a), in which 26 tissue sections from the colorectal carcinoma specimen are divided into K groups. Each time one group is designated as the testing set, while the other groups are regarded as the training set. This is repeated K times, ensuring that each group can serve as the testing set at least once, and providing a comprehensive evaluation of model performance.

In the MSI data of colorectal adenocarcinoma dataset, K is selected as 5 for the cross-validation (same as reference [17]). During each iteration of the 5-fold cross-validation, the data from 4 randomly selected groups, comprised of 20 tissue sections with a total of 93,482 spectra, are used to train Atnal. The training process of Atnal becomes convergent after approximately 15 epochs, as depicted in Fig. 4b. The total running time for this convergence is approximately 1.5 min. Based on the encoded features (Supplementary Fig. 6) derived by the encoder of Atnal, the original spectra are reconstructed with an overall mean squared training error of $1.41 \times 10^{-7}$.

Once the model training is completed, its performance is further evaluated by using the remaining 6 tissue sections with 29,006 spectra (testing set). It takes approximately 4 s for the encoder of Atnal to predict the encoded features and another 3 s for the decoder to reconstruct the data.

In Fig. 5a and b, the distribution of Atnal’s MSE across *m/z* values and ion intensities in the testing set is presented. The results indicate that Atnal achieves low-error reconstruction across all ion peaks, without significant bias. However, for high-intensity ion peaks, the reconstruction error tends to increase, showing a significant correlation (Pearson correlation coefficient = 0.88). This observation aligns with prior knowledge that low-intensity ion peaks should not be neglected during reconstruction.

The strong resemblance observed between the original and reconstructed spectra, as shown in Supplementary Figs. 3 and 4, indicates that the encoder successfully captures the intricate features present in the nonlinear manifold of the original high-dimensional data.

The encoded features, in Supplementary Fig. 7, represent a 256-dimensional nonlinear embedding of the original high-dimensional testing data. The overall mean square testing error between the reconstructed and original data is $2.1 \times 10^{-7}$. The testing results, 5 GMM clusters, of the encoded features, carry significant implication: 2 of 5 GMM clusters associate with tumor and connective tissue. This implication is consistent with the results of previous studies [41] [22] and images of the H&E stained tissue sections, see Fig. 5e (H&E histology) and Supplementary Fig. 8.

The encoded features are then clustered by GMM (k = 5), forming five clusters, and two of them (cluster 3 and cluster 5) show a high degree of consistency with histological evaluation (H&E) as illustrated in Fig. 5e (H&E histology) and Supplementary Fig. 9. These two clusters correlate with the ions at *m/z* 720.4954 (Supplementary Fig. 15) and *m/z* 766.53, of which the Pearson correlation values are 0.7761 and 0.746, respectively. Moreover, this result is consistent with the observation that the intensities of the ions at *m/z* 720.49 and *m/z* 766.53 are particularly high in tumor and connective tissue, respectively.

14 *m/z* values are discovered to be strongly correlated with colorectal carcinoma. As checked with the Human Metabolome Database (HMDB), the LIPID MAPS®Structure Database (LMSD) and MetaboAnalyst database, 9 out of 14 have putative annotations from database searches. For instance, *m/z* 858.5260627 has a putative annotation suggesting compounds belonging to the class of phosphatidylserines with a tolerance window of 5 ppm. These putative annotations are speculated to be related to the vigorous phospholipid metabolism of colorectal cancer cells. Nevertheless, there is no relevant report on phosphatidylserine compounds as colorectal cancer markers. For more details on the tentative annotations of these compounds, see Supplementary Table 2, 3.

## Discussion

In this study, a novel method called Atnal is developed for analyzing MSI data collected from different types of human tissue. It can be used to reveal heterogeneity in cancer tissue, extract spatial molecular patterns, and propose putative biomarker candidates for further validation in cancer research and clinical diagnosis. In the following, we discuss the details of the training and application (dimension reduction, clustering and correlation analysis) of Atnal.

**Fig. 6:**
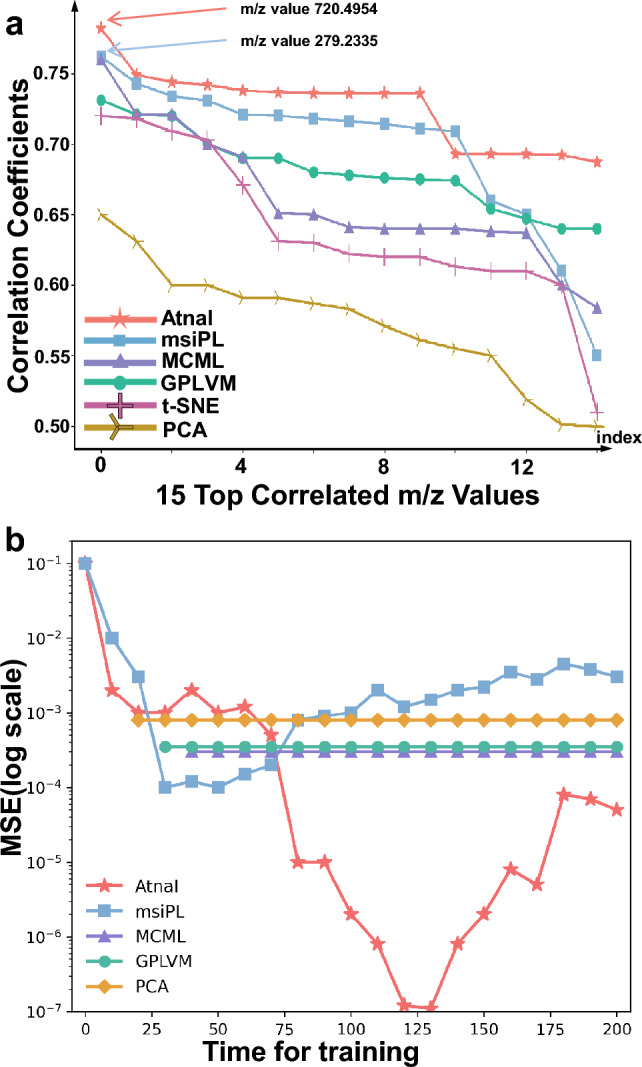
Quantitative comparative analysis of model performance (using colorectal adenocarcinoma dataset). **a** The top-ranked *m/z* values with the highest Pearson correlation coefficients with the cancer cluster; Baselines include: msiPL [17], MCML [42], GPLVM [43], t-SNE, and PCA. This subplot is presented as supportive interpretability evidence rather than a cross-method quantitative benchmark. **b** The logarithmic mean squared errors between original and reconstructed MSI data computed via various techniques. Here, t-SNE in subplot (**a**) is not included in subplot (**b**) because its low-dimensional results are mathematically irreversible.

**Table 2:** MSEs for MSI data reconstruction

Dataset	MCML	GPLVM	PCA	msiPL	Atnal
	[42]	[43]		[17]	(Ours)
Prostate	$2.4 \times 10^{-3}$	$1.1 \times 10^{-4}$	$2.1 \times 10^{-4}$	$6.9 \times 10^{-5}$	$7.0 \times 10^{-7}$
Colorectal	$2.1\times 10^{-4}$	$2.7\times 10^{-4}$	$8.1\times 10^{-4}$	$8.6 \times 10^{-5}$	$2.1 \times 10^{-7}$

**Table 3:** Ablation of Atnal with colorectal carcinoma dataset

Experimental condition	Loss	
	Training	Testing
Base	$1.567 \times 10^{-7}$	$2.262 \times 10^{-7}$
Base + Residual	$2.022 \times 10^{-7}$	$2.863 \times 10^{-7}$
Base + Attention	$4.201 \times 10^{-7}$	$8.113 \times 10^{-7}$
Base + Attention + Residual	$\mathbf {1.414 \times 10^{-7}}$	$\mathbf {2.119 \times 10^{-7}}$

*Training* During the training, unsupervised learning is employed to extract prior information from the raw data; ReLU activation functions are extensively adopted to introduce non-linearity; and Softmax activation functions are used in the final output layer to ensure the reconstructed data values have the same range as the normalized input, that is, from 0 to 1. The consistency of the value range between the input and output layers is crucial for minimizing the loss function expressed by Eq. 10. Adopting the learning rate decay in unsupervised learning, Atnal shows strong generalization and excellent performance on reconstructing original data, and avoids overfitting the training data, as shown in Fig. 4b. As an automated manifold learning framework, Atnal can accelerate the identification of biologically relevant ions and reduce the biases introduced by subjective factors. The loss function is given by the categorical cross-entropy that assigns higher weights to non-zero data in the spectral data and makes Atnal more sensitive to the reconstruction of the original data. Atnal uses mini-batch training, which allows loading only a small portion of the spectral data into the graphics memory. This enables the Graphics Processing Units (GPUs) to process large and complex datasets, and empowers Atnal to conduct single-precision floating-point training on a workstation with 64 GB of memory and 8 GB of graphics memory.

As the kernel of Atnal, the attention mechanism simulates the human attention mechanism, and assists Atnal in focusing on relevant parts of the input sequence in the spectra by treating the intensity at different *m/z* values as the features of these spectra. Without any positional information, the attention matrices between different spectra are constructed to discover the intrinsic correlations among these spectra. In the Attention Block ($h_3$), the keys, values, and queries (mentioned in Eq. 4) are all derived from the output of the previous layer $MLP_e$ ($h_2$) in the encoder. As a normal module used in deep neural networks, the residual connection module provides a direct flow of information within $h_3$.

To provide a comprehensive evaluation of Atnal, several representative baselines are compared on the colorectal adenocarcinoma dataset, including classical dimensionality-reduction methods (PCA and t-SNE), the Gaussian Process Latent Variable Model (GPLVM [43]), and recent autoencoder-based approaches, namely msiPL [17] and MCML (Manifold-Constrained Metric Learning[42]). The low-dimensional representations generated by the above methods are all clustered using GMM (k = 5). The hyperparameter settings for these methods are summarized as follows.

For PCA, the first 256 principal components are retained. Given the large number of spectra in the colorectal adenocarcinoma dataset (122,488 non-zero spectra), incremental computation is adopted using sklearn.decomposition.IncrementalPCA to accommodate the large-scale data.

For t-SNE, the standard implementation (sklearn.manifold.TSNE) becomes prohibitively memory-intensive on our large MSI dataset, because it requires computing and storing dense pairwise affinities. Therefore, PCA is first applied to reduce the spectra to 1024 dimensions as a preprocessing step, and then t-SNE is performed using openTSNE with n_components=24 and perplexity=40. We do not enforce a 256-dimensional t-SNE embedding (to match Atnal) because t-SNE is primarily intended for low-dimensional embeddings; higher target dimensions often yield non-identifiable, quasi-equivalent optima, increased computational cost, and limited additional structure for downstream clustering.

For GPLVM [43], the latent dimensionality is set to 256 to align with Atnal. Following the original formulation, an "RBF kernel + white-noise" covariance function is employed: the RBF kernel captures nonlinear similarity among nearby samples in the latent space, while the white-noise term adds a small diagonal jitter to model observation noise and improves numerical stability. Kernel hyperparameters are optimized via gradient-based learning (maximum 1000 iterations; convergence tolerance $10^{-6}$).

The msiPL model adopts a variational autoencoder (VAE) architecture consisting of an input layer, three hidden layers, and an output layer. In the original study, the neuron configuration is *m/z* bins–512–5–512–*m/z* bins. In this work, the bottleneck dimensionality is increased from 5 to 256 to match Atnal.

For MCML (Manifold-Constrained Metric Learning[42]), the original joint objective combining distance preservation and reconstruction is followed:

12 $$\begin{aligned} L = \lambda _1 L_{\textrm{Dist}} + \lambda _2 L_{\textrm{Recon}} . \end{aligned}$$

The principal latent dimensionalities associated with $\textrm{Dist}$ and $\textrm{Recon}$ are both set to 256 to align with Atnal. The weights $\lambda _1$ and $\lambda _2$ are set to 0.2 and 0.8, respectively, to balance geometric fidelity and reconstruction quality.

As shown in Fig. 6b and Table 2, Atnal has lower MSE than PCA, GPLVM and other encoder-decoder-based methods (msiPL and MCML) by at least two orders of magnitude. Additionally, the loss values of msiPL and Atnal in Fig. 6b exhibit an upward trend after a certain period. All clustering results (including Atnal and all baseline methods) on the colorectal adenocarcinoma dataset are shown in Supplementary Figs. 2, 10, 11, 12, 13, and 14. Only the clusters most strongly associated with cancer and connective tissue are displayed.

To test the functions of the attention module and the residual connection module, we conduct an ablation experiment. As shown in Table 3, using only one of the two modules results in a varying degree of decrease in model performance. Moreover, as a comparison, when we incorporate both the attention module and the residual connection module as submodules in Atnal, we can achieve optimal results.

The following considerations explain the ablation outcomes. The attention module enables the model to learn the weights of different positions in the input, and allows for focusing on the spectra located in the positions with high weights and reducing the interference from the information with low weights. But, relying only on the attention module (without residual module) may lead to an excessive attention on some certain positions or features. Similarly, the residual connection module may facilitate direct information propagation within networks and respond to challenges from the gradient vanishing and exploding, so as to reduce information loss in deep networks on one hand. On the other hand, only using the residual connection module may introduce too much information flow, which results in an overreliance on previous layers and limits model stability and expressive capacity. As seen, integrating the residual connection module and the attention module contribute to a balance between these advantages and disadvantages, which benefits both the information transmission and selection, thus enhancing the overall performance.

*Application* After training, the original high-dimensional spectral data is encoded into low-dimensional features by the encoder of Atnal. The data complexity (for subsequent analysis) is reduced from tens of thousands of dimensions to 256 dimensions (Table 1). This avoids the "curse of dimensionality" problem commonly encountered in traditional machine learning methods.

The obtained encoded features are then fed into the GMM clustering algorithm. Clustering results in Fig. 5e show spectra corresponding to cancer regions (pathologist annotation) grouping coherently and distinctly from non-cancer spectra. Based on this, correlations between each *m/z* dimension and the cancer cluster are computed, yielding *m/z* values with high association to cancer regions (Fig. 6a). Although Fig. 5e (Clustering result column) reveals ion interference in non-blank background regions, the two selected clusters (Selected clusters column) effectively segregate these interfering areas, demonstrating Atnal’s robustness against noise and its discriminative capability.

These identified *m/z* values can indicate potential biomarkers and be further used to search for corresponding substances in databases. In Fig. 5d, we conduct cross-validation analyses and obtain a list of the top 100 *m/z* values based on their correlations. Among these values, there are 121 unique *m/z* peaks. Remarkably, 80 (66% of 121) are identified as stable, and consistently detected across all cross-validation analyses.

Beyond MSI, Atnal has been preliminarily explored for other modalities, such as CT-based phenotyping in chronic obstructive pulmonary disease (COPD), demonstrating promising generalizability. The corresponding implementation will be organized and released in the same GitHub repository upon curation.

## Conclusion

In this study, we present Atnal, an unsupervised manifold learning framework that integrates a diffusion-based encoder with autoregressive modeling to address the challenges of high-dimensional MSI data analysis. By effectively reducing dimensionality while preserving critical molecular patterns, Atnal enables robust clustering, visualization, and biomarker discovery. Applied to prostate cancer and colorectal adenocarcinoma datasets, Atnal not only delineates tumor regions with high accuracy but also aids in revealing key metabolic markers with Pearson correlation coefficients up to 0.79, demonstrating its potential for advancing precision oncology.

The diffusion-based encoder with autoregressive modeling is a key innovation of Atnal, allowing for noise reduction and the extraction of meaningful features from noisy MSI data. This architecture ensures that essential molecular patterns are retained, enabling accurate reconstruction and interpretation of MSI data. Compared to existing methods, Atnal achieves superior performance in both data reconstruction and the exploration of potential biomarkers.

Looking forward, Atnal represents a significant step toward the integration of advanced machine learning techniques in precision oncology. Future work will focus on optimizing the computational efficiency of the model and extending its application to other omics datasets, further enhancing its utility in clinical and research settings.

## Supplementary Information

Below is the link to the electronic supplementary material.

Supplementary Material 1

## Data Availability

The source code will be available at GitHub (https://github.com/AmFe-GH/Atnal). As previously mentioned, the used datasets are accessible at http://gigadb.org/dataset/100131 and https://doi.org/10.21228/M8BM4Q, respectively.
