# Supplementary material for: Towards precision oncology: unsupervised manifold learning for spatial molecular profiling in cancer tissues
Source: BMC Bioinformatics. 2026 May 15;27:154. doi: 10.1186/s12859-026-06462-8 (PMC13390296; doi:10.1186/s12859-026-06462-8)
Supplement: Supplementary file 1 — Supplementary Material 1 [file 12859_2026_6462_MOESM1_ESM.pdf]

Supplementary Information
Towards Precision Oncology: Unsupervised Manifold Learning for Spatial Molecular Profiling in Cancer Tissues

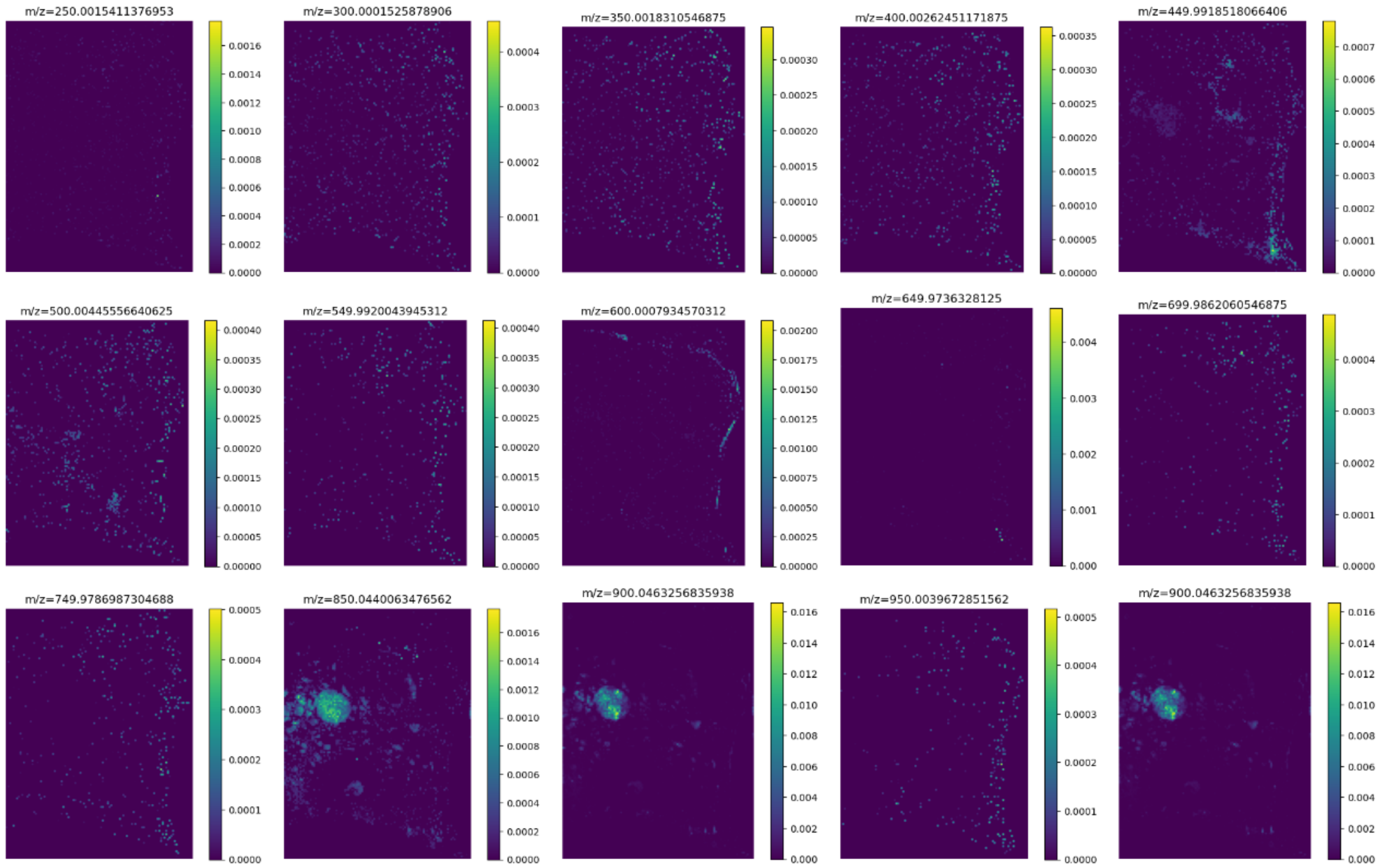

Figure 1: **Several patterns of MSI data from prostate cancer tissues**. It is readily apparent that at most m/z channels, the intensity of MSI is faint and disorganized. Such raw data cannot be directly utilized for subsequent analysis. Conventional approaches involve dimensionality reduction and manual selection of specific m/z channels.

m/z	compound	formula	structural formula	class	pathway	adduct	benchmark m/z	ppm difference	database	HMDB-ID	database-ID
774.5983	PC(15:020:1(11Z))	1-Pentadecanoyl-2-eicosenoyl-sn-glycero-3-phosphocholine	$C_{43}H_{84}NO_8P$	Phosphatidylcholines	1. Phosphatidylcholine Biosynthesis 2. Phosphatidylethanolamine Biosynthesis	M+H	774.6007312	3.138649245	HMDB	HMDB07945	HMDB07945
774.5983	PC(20:1(11Z)15:0)	1-Eicosenoyl-2-pentadecanoyl-sn-glycero-3-phosphocholine	$C_{43}H_{84}NO_8P$	Phosphatidylcholines	1. Phosphatidylcholine Biosynthesis 2. Phosphatidylethanolamine Biosynthesis	M+H	774.6007312	3.138649245	HMDB	HMDB08297	HMDB08297
774.5983	PE(14:024:1(15Z))	1-Myristoyl-2-nervonoyl-sn-glycero-3-phosphoethanolamine	$C_{43}H_{84}NO_8P$	Phosphatidylethanolamines	1. Phosphatidylcholine Biosynthesis 2. Phosphatidylethanolamine Biosynthesis	M+H	774.6007312	3.138649245	HMDB	HMDB08849	HMDB08849
774.5983	PE(14:1(9Z)24:0)	1-Myristoleoyl-2-lignoceroyl-sn-glycero-3-phosphoethanolamine	$C_{43}H_{84}NO_8P$	Phosphatidylethanolamines	1. Phosphatidylcholine Biosynthesis 2. Phosphatidylethanolamine Biosynthesis	M+H	774.6007312	3.138649245	HMDB	HMDB08881	HMDB08881
774.5983	PE(16:022:1(13Z))	1-Palmitoyl-2-erucoyl-sn-glycero-3-phosphoethanolamine	$C_{43}H_{84}NO_8P$	Phosphatidylethanolamines	1. Phosphatidylcholine Biosynthesis 2. Phosphatidylethanolamine Biosynthesis	M+H	774.6007312	3.138649245	HMDB	HMDB08941	HMDB08941
774.5983	PE(16:1(9Z)22:0)	1-Palmitoleoyl-2-behenoyl-sn-glycero-3-phosphoethanolamine	$C_{43}H_{84}NO_8P$	Phosphatidylethanolamines	1. Phosphatidylcholine Biosynthesis 2. Phosphatidylethanolamine Biosynthesis	M+H	774.6007312	3.138649245	HMDB	HMDB08973	HMDB08973
774.5983	PE(18:020:1(11Z))	1-Stearoyl-2-eicosenoyl-sn-glycero-3-phosphoethanolamine	$C_{43}H_{84}NO_8P$	Phosphatidylethanolamines	1. Phosphatidylcholine Biosynthesis 2. Phosphatidylethanolamine Biosynthesis	M+H	774.6007312	3.138649245	HMDB	HMDB08999	HMDB08999
774.5983	PE(18:1(11Z)20:0)	1-Vaccenoyl-2-arachidonyl-sn-glycero-3-phosphoethanolamine	$C_{43}H_{84}NO_8P$	Phosphatidylethanolamines	1. Phosphatidylcholine Biosynthesis 2. Phosphatidylethanolamine Biosynthesis	M+H	774.6007312	3.138649245	HMDB	HMDB09031	HMDB09031
774.5983	PE(18:1(9Z)20:0)	1-Oleoyl-2-arachidonyl-sn-glycero-3-phosphoethanolamine	$C_{43}H_{84}NO_8P$	Phosphatidylethanolamines	1. Phosphatidylcholine Biosynthesis 2. Phosphatidylethanolamine Biosynthesis	M+H	774.6007312	3.138649245	HMDB	HMDB09064	HMDB09064
774.5983	PE(20:018:1(11Z))	1-Arachidonyl-2-vaccenoyl-sn-glycero-3-phosphoethanolamine	$C_{43}H_{84}NO_8P$	Phosphatidylethanolamines	1. Phosphatidylcholine Biosynthesis 2. Phosphatidylethanolamine Biosynthesis	M+H	774.6007312	3.138649245	HMDB	HMDB09223	HMDB09223
774.5983	PE(20:018:1(9Z))	1-Arachidonyl-2-oleoyl-sn-glycero-3-phosphoethanolamine	$C_{43}H_{84}NO_8P$	Phosphatidylethanolamines	1. Phosphatidylcholine Biosynthesis 2. Phosphatidylethanolamine Biosynthesis	M+H	774.6007312	3.138649245	HMDB	HMDB09224	HMDB09224
774.5983	PE(20:1(11Z)18:0)	1-Eicosenoyl-2-stearoyl-sn-glycero-3-phosphoethanolamine	$C_{43}H_{84}NO_8P$	Phosphatidylethanolamines	1. Phosphatidylcholine Biosynthesis 2. Phosphatidylethanolamine Biosynthesis	M+H	774.6007312	3.138649245	HMDB	HMDB09255	HMDB09255
774.5983	PE(22:016:1(9Z))	1-Behenoyl-2-palmitoleoyl-sn-glycero-3-phosphoethanolamine	$C_{43}H_{84}NO_8P$	Phosphatidylethanolamines	1. Phosphatidylcholine Biosynthesis 2. Phosphatidylethanolamine Biosynthesis	M+H	774.6007312	3.138649245	HMDB	HMDB09485	HMDB09485
774.5983	PE(22:1(13Z)16:0)	1-Erucoyl-2-palmitoyl-sn-glycero-3-phosphoethanolamine	$C_{43}H_{84}NO_8P$	Phosphatidylethanolamines	1. Phosphatidylcholine Biosynthesis 2. Phosphatidylethanolamine Biosynthesis	M+H	774.6007312	3.138649245	HMDB	HMDB09517	HMDB09517
774.5983	PE(24:014:1(9Z))	1-lignoceroyl-2-myristoleoyl-sn-glycero-3-phosphoethanolamine	$C_{43}H_{84}NO_8P$	Phosphatidylethanolamines	1. Phosphatidylcholine Biosynthesis 2. Phosphatidylethanolamine Biosynthesis	M+H	774.6007312	3.138649245	HMDB	HMDB09713	HMDB09713
774.5983	PE(24:1(15Z)14:0)	1-Nervonoyl-2-myristoyl-sn-glycero-3-phosphoethanolamine	$C_{43}H_{84}NO_8P$	Phosphatidylethanolamines	1. Phosphatidylcholine Biosynthesis 2. Phosphatidylethanolamine Biosynthesis	M+H	774.6007312	3.138649245	HMDB	HMDB09745	HMDB09745
884.5723	NA										
845.6712	PE(18:1(11Z)24:1(15Z))	1-Vaccenoyl-2-nervonoyl-sn-glycero-3-phosphoethanolamine	$C_{47}H_{90}NO_8P$	Phosphatidylethanolamines	1. Phosphatidylcholine Biosynthesis 2. Phosphatidylethanolamine Biosynthesis	M+NH4	845.6742284	3.581047995	HMDB	HMDB09047	HMDB09047
845.6712	PE(18:1(9Z)24:1(15Z))	1-Oleoyl-2-nervonoyl-sn-glycero-3-phosphoethanolamine	$C_{47}H_{90}NO_8P$	Phosphatidylethanolamines	1. Phosphatidylcholine Biosynthesis 2. Phosphatidylethanolamine Biosynthesis	M+NH4	845.6742284	3.581047995	HMDB	HMDB09080	HMDB09080
845.6712	PE(18:2(9Z12Z)24:0)	1-Linoleoyl-2-lignoceroyl-sn-glycero-3-phosphoethanolamine	$C_{47}H_{90}NO_8P$	Phosphatidylethanolamines	1. Phosphatidylcholine Biosynthesis 2. Phosphatidylethanolamine Biosynthesis	M+NH4	845.6742284	3.581047995	HMDB	HMDB09112	HMDB09112
845.6712	PE(20:022:2(13Z16Z))	1-Arachidonyl-2-docosadienoyl-sn-glycero-3-phosphoethanolamine	$C_{47}H_{90}NO_8P$	Phosphatidylethanolamines	1. Phosphatidylcholine Biosynthesis 2. Phosphatidylethanolamine Biosynthesis	M+NH4	845.6742284	3.581047995	HMDB	HMDB09239	HMDB09239
845.6712	PE(20:1(11Z)22:1(13Z))	1-Eicosenoyl-2-erucoyl-sn-glycero-3-phosphoethanolamine	$C_{47}H_{90}NO_8P$	Phosphatidylethanolamines	1. Phosphatidylcholine Biosynthesis 2. Phosphatidylethanolamine Biosynthesis	M+NH4	845.6742284	3.581047995	HMDB	HMDB09271	HMDB09271
845.6712	PE(20:2(11Z14Z)22:0)	1-Eicosadienoyl-2-behenoyl-sn-glycero-3-phosphoethanolamine	$C_{47}H_{90}NO_8P$	Phosphatidylethanolamines	1. Phosphatidylcholine Biosynthesis 2. Phosphatidylethanolamine Biosynthesis	M+NH4	845.6742284	3.581047995	HMDB	HMDB09303	HMDB09303
845.6712	PE(22:020:2(11Z14Z))	1-Behenoyl-2-eicosadienoyl-sn-glycero-3-phosphoethanolamine	$C_{47}H_{90}NO_8P$	Phosphatidylethanolamines	1. Phosphatidylcholine Biosynthesis 2. Phosphatidylethanolamine Biosynthesis	M+NH4	845.6742284	3.581047995	HMDB	HMDB09495	HMDB09495
845.6712	PE(22:1(13Z)20:1(11Z))	1-Erucoyl-2-eicosenoyl-sn-glycero-3-phosphoethanolamine	$C_{47}H_{90}NO_8P$	Phosphatidylethanolamines	1. Phosphatidylcholine Biosynthesis 2. Phosphatidylethanolamine Biosynthesis	M+NH4	845.6742284	3.581047995	HMDB	HMDB09527	HMDB09527
845.6712	PE(22:2(13Z16Z)20:0)	1-Docosadienoyl-2-arachidonyl-sn-glycero-3-phosphoethanolamine	$C_{47}H_{90}NO_8P$	Phosphatidylethanolamines	1. Phosphatidylcholine Biosynthesis 2. Phosphatidylethanolamine Biosynthesis	M+NH4	845.6742284	3.581047995	HMDB	HMDB09559	HMDB09559
845.6712	PE(24:018:2(9Z12Z))	1-Lignoceroyl-2-linoleoyl-sn-glycero-3-phosphoethanolamine	$C_{47}H_{90}NO_8P$	Phosphatidylethanolamines	1. Phosphatidylcholine Biosynthesis 2. Phosphatidylethanolamine Biosynthesis	M+NH4	845.6742284	3.581047995	HMDB	HMDB09720	HMDB09720
845.6712	PE(24:1(15Z)18:1(11Z))	1-nervonoyl-2-vaccenoyl-sn-glycero-3-phosphoethanolamine	$C_{47}H_{90}NO_8P$	Phosphatidylethanolamines	1. Phosphatidylcholine Biosynthesis 2. Phosphatidylethanolamine Biosynthesis	M+NH4	845.6742284	3.581047995	HMDB	HMDB09751	HMDB09751
845.6712	PE(24:1(15Z)18:1(9Z))	1-nervonoyl-2-oleoyl-sn-glycero-3-phosphoethanolamine	$C_{47}H_{90}NO_8P$	Phosphatidylethanolamines	1. Phosphatidylcholine Biosynthesis 2. Phosphatidylethanolamine Biosynthesis	M+NH4	845.6742284	3.581047995	HMDB	HMDB09752	HMDB09752
775.6025	NA										
760.5827	PC(14:020:1(11Z))	1-Myristoyl-2-eicosenoyl-sn-glycero-3-phosphocholine	$C_{42}H_{82}NO_8P$	Phosphatidylcholines	1. Phosphatidylcholine Biosynthesis 2. Phosphatidylethanolamine Biosynthesis	M+H	760.5850811	3.130616231	HMDB	HMDB07879	HMDB07879
760.5827	PC(14:1(9Z)20:0)	1-Myristoleoyl-2-arachidonyl-sn-glycero-3-phosphocholine	$C_{42}H_{82}NO_8P$	Phosphatidylcholines	1. Phosphatidylcholine Biosynthesis 2. Phosphatidylethanolamine Biosynthesis	M+H	760.5850811	3.130616231	HMDB	HMDB07911	HMDB07911
760.5827	PC(16:018:1(11Z))	1-Palmitoyl-2-vaccenoyl-sn-glycero-3-phosphocholine	$C_{42}H_{82}NO_8P$	Phosphatidylcholines	1. Phosphatidylcholine Biosynthesis 2. Phosphatidylethanolamine Biosynthesis	M+H	760.5850811	3.130616231	HMDB	HMDB07971	HMDB07971
760.5827	PC(16:018:1(9Z))	1-Hexadecanoyl-2-oleoyl-sn-glycero-3-phosphocholine	$C_{42}H_{82}NO_8P$	Phosphatidylcholines	1. Phosphatidylcholine Biosynthesis 2. Phosphatidylethanolamine Biosynthesis	M+H	760.5850811	3.130616231	HMDB	HMDB07972	HMDB07972
760.5827	PC(16:1(9Z)18:0)	1-(9Z-Hexadecenoyl)-2-octadecanoyl-sn-glycero-3-phosphocholine	$C_{42}H_{82}NO_8P$	Phosphatidylcholines	1. Phosphatidylcholine Biosynthesis 2. Phosphatidylethanolamine Biosynthesis	M+H	760.5850811	3.130616231	HMDB	HMDB08003	HMDB08003
760.5827	PC(18:016:1(9Z))	1-Stearoyl-2-palmitoleoyl-sn-glycero-3-phosphocholine	$C_{42}H_{82}NO_8P$	Phosphatidylcholines	1. Phosphatidylcholine Biosynthesis 2. Phosphatidylethanolamine Biosynthesis	M+H	760.5850811	3.130616231	HMDB	HMDB08035	HMDB08035
760.5827	PC(18:1(11Z)16:0)	1-Vaccenoyl-2-palmitoyl-sn-glycero-3-phosphocholine	$C_{42}H_{82}NO_8P$	Phosphatidylcholines	1. Phosphatidylcholine Biosynthesis 2. Phosphatidylethanolamine Biosynthesis	M+H	760.5850811	3.130616231	HMDB	HMDB08067	HMDB08067
760.5827	PC(18:1(9Z)16:0)	1-Palmotoyl-2-oleoylglycero-3-phosphocholine	$C_{42}H_{82}NO_8P$	Phosphatidylcholines	1. Phosphatidylcholine Biosynthesis 2. Phosphatidylethanolamine Biosynthesis	M+H	760.5850811	3.130616231	HMDB	HMDB08100	HMDB08100
760.5827	PC(20:014:1(9Z))	1-arachidonyl-2-myristoleoyl-sn-glycero-3-phosphocholine	$C_{42}H_{82}NO_8P$	Phosphatidylcholines	1. Phosphatidylcholine Biosynthesis 2. Phosphatidylethanolamine Biosynthesis	M+H	760.5850811	3.130616231	HMDB	HMDB08263	HMDB08263
760.5827	PC(20:1(11Z)14:0)	1-Eicosenoyl-2-myristoyl-sn-glycero-3-phosphocholine	$C_{42}H_{82}NO_8P$	Phosphatidylcholines	1. Phosphatidylcholine Biosynthesis 2. Phosphatidylethanolamine Biosynthesis	M+H	760.5850811	3.130616231	HMDB	HMDB08295	HMDB08295
760.5827	PE(15:022:1(13Z))	1-Pentadecanoyl-2-erucoyl-sn-glycero-3-phosphoethanolamine	$C_{42}H_{82}NO_8P$	Phosphatidylethanolamines	1. Phosphatidylcholine Biosynthesis 2. Phosphatidylethanolamine Biosynthesis	M+H	760.5850811	3.130616231	HMDB	HMDB08908	HMDB08908
760.5827	PE(22:1(13Z)15:0)	1-Erucoyl-2-pentadecanoyl-sn-glycero-3-phosphoethanolamine	$C_{42}H_{82}NO_8P$	Phosphatidylethanolamines	1. Phosphatidylcholine Biosynthesis 2. Phosphatidylethanolamine Biosynthesis	M+H	760.5850811	3.130616231	HMDB	HMDB09516	HMDB09516
761.5867	NA	Termitomycesphin A	$C_{41}H_{77}NO_{10}$	Neutral glycosphingolipids	NA	M+NH4	761.588573	2.459333118	Lipidmaps	NA	LMSP01080015
829.6375	NA									NA	
863.6799	3-O-(6-O-(11Z14Z17Z-eicosatrienoyl)-beta-D-glucopyranosyl)-stigmast-522E-dien-3beta-ol	3-O-(6'-O-(11Z,14Z,17Z-eicosatrienoyl)-beta-D-glucopyranosyl)-stigmast-5,22E-dien-3beta-ol	$C_{55}H_{90}O_7$	Sterols	NA	M+H	863.675933	4.593157976	Lipidmaps	NA	LMST01040233
803.6224	NA	NA	NA		NA					NA	
504.3485	NA	NA	NA		NA					NA	
846.677	NA	NA	NA		NA					NA	
762.5862	N-(docosanoyl)-1-beta-glucosyl-4E6E-pentadecasphingadienine	N-(docosanoyl)-1-beta-glucosyl-4E,6E-pentadecasphingadienine	$C_{43}H_{81}NO_8$	Neutral glycosphingolipids	NA	M+Na	762.585438	0.999232298	Lipidmaps	NA	LMSP0501AA60
864.6829	NA	NA	NA		NA					NA	
253.5388	NA	NA	NA		NA					NA	
804.6077	1-hexadecyl-2-(11Z-docosenoyl)-glycero-3-phosphoserine	1-hexadecyl-2-(11Z-docosenoyl)-glycero-3-phosphoserine	$C_{44}H_{86}NO_9P$	Glycerophospholipids	NA	M+H	804.611299	4.472967263	Lipidmaps	NA	LMGP03020015
804.6077	1-octadecyl-2-(11Z-eicosenoyl)-glycero-3-phosphoserine	1-octadecyl-2-(11Z-eicosenoyl)-glycero-3-phosphoserine	$C_{44}H_{86}NO_9P$	Glycerophospholipids	NA	M+H	804.611299	4.472967263	Lipidmaps	NA	LMGP03020034
804.6077	1-eicosyl-2-(9Z-octadecenoyl)-glycero-3-phosphoserine	1-eicosyl-2-(9Z-octadecenoyl)-glycero-3-phosphoserine	$C_{44}H_{86}NO_9P$	Glycerophospholipids	NA	M+H	804.611299	4.472967263	Lipidmaps	NA	LMGP03020052
804.6077	1-(1Z-hexadecenyl)-2-docosanoyl-glycero-3-phosphoserine	1-(1Z-hexadecenyl)-2-docosanoyl-glycero-3-phosphoserine	$C_{44}H_{86}NO_9P$	Glycerophospholipids	NA	M+H	804.611299	4.472967263	Lipidmaps	NA	LMGP03030024
804.6077	1-(1Z-octadecenyl)-2-eicosanoyl-glycero-3-phosphoserine	1-(1Z-octadecenyl)-2-eicosanoyl-glycero-3-phosphoserine	$C_{44}H_{86}NO_9P$	Glycerophospholipids	NA	M+H	804.611299	4.472967263	Lipidmaps	NA	LMGP03030046
804.6077	1-(1Z-eicosenyl)-2-octadecanoyl-glycero-3-phosphoserine	1-(1Z-eicosenyl)-2-octadecanoyl-glycero-3-phosphoserine	$C_{44}H_{86}NO_9P$	Glycerophospholipids	NA	M+H	804.611299	4.472967263	Lipidmaps	NA	LMGP03030067
804.6077	1-octadecyl-2-(8Z11Z14Z-eicosatrienoyl)-glycero-3-phospho-(1-sn-glycerol)	1-octadecyl-2-(8Z,11Z,14Z-eicosatrienoyl)-glycero-3-phospho-(1'-sn-glycerol)	$C_{44}H_{86}NO_9P$	Glycerophospholipids	NA	M+NH4	804.611297	4.470481602	Lipidmaps	NA	LMGP04020036
804.6077	1-eicosyl-2-(6Z9Z12Z-octadecatrienoyl)-glycero-3-phospho-(1-sn-glycerol)	1-eicosyl-2-(6Z,9Z,12Z-octadecatrienoyl)-glycero-3-phospho-(1'-sn-glycerol)	$C_{44}H_{86}NO_9P$	Glycerophospholipids	NA	M+NH4	804.611297	4.470481602	Lipidmaps	NA	LMGP04020054
804.6077	1-eicosyl-2-(9Z12Z15Z-octadecatrienoyl)-glycero-3-phospho-(1-sn-glycerol)	1-eicosyl-2-(9Z,12Z,15Z-octadecatrienoyl)-glycero-3-phospho-(1'-sn-glycerol)	$C_{44}H_{86}NO_9P$	Glycerophospholipids	NA	M+NH4	804.611297	4.470481602	Lipidmaps	NA	LMGP04020055
804.6077	1-(1Z-hexadecenyl)-2-(13Z16Z-docosadienoyl)-glycero-3-phospho-(1-sn-glycerol)	1-(1Z-hexadecenyl)-2-(13Z,16Z-docosadienoyl)-glycero-3-phospho-(1'-sn-glycerol)	$C_{44}H_{86}NO_9P$	Glycerophospholipids	NA	M+NH4	804.611297	4.470481602	Lipidmaps	NA	LMGP04030026
804.6077	1-(1Z-octadecenyl)-2-(11Z14Z-eicosadienoyl)-glycero-3-phospho-(1-sn-glycerol)	1-(1Z-octadecenyl)-2-(11Z,14Z-eicosadienoyl)-glycero-3-phospho-(1'-sn-glycerol)	$C_{44}H_{86}NO_9P$	Glycerophospholipids	NA	M+NH4	804.611297	4.470481602	Lipidmaps	NA	LMGP04030048
804.6077	1-(1Z-eicosenyl)-2-(9Z12Z-octadecadienoyl)-glycero-3-phospho-(1-sn-glycerol)	1-(1Z-eicosenyl)-2-(9Z,12Z-octadecadienoyl)-glycero-3-phospho-(1'-sn-glycerol)	$C_{44}H_{86}NO_9P$	Glycerophospholipids	NA	M+NH4	804.611297	4.470481602	Lipidmaps	NA	LMGP04030069

Table 1: **Identified potential biomarkers of prostate cancer**. Among the 15 m/z values with top-ranked correlation coefficients, 7 of them are successfully identified within a tolerance window of 5 ppm. For example, the m/z value 774.5983 indicates 16 potential phosphatidylcholine and phosphatidylethanolamine metabolites. The m/z 804.6077 is identified as 12 potential compounds that belong to the class of glycerophospholipids and previously identified in prostate cancer samples in the database, Metabolomics Workbench (https://www.metabolomicsworkbench.org), with study IDs ST000784 and ST001133. As for the m/z 762.5862, it is identified as a neutral glycosphingolipids compound.

m/z	compound	formula	structural formula	class	pathway	adduct	benchmark m/z	ppm difference	database	HMDB-ID	database-ID
858.5260627	1-(5Z8Z11Z14Z-eicosatetraenoyl)-2-(7Z10Z13Z16Z-docosatetraenoyl)-glycero-3-phosphoserine	1-(5Z,8Z,11Z,14Z-eicosatetraenoyl)-2-(7Z,10Z,13Z,16Z-docosatetraenoyl)-glycero-3-phosphoserine	$C_{48}H_{78}NO_{10}P$	Phosphatidylserines	(1) Phosphatidylcholine Biosynthesis; (2)Phosphatidylethanolamine Biosynthesis	M-H	858.529062	3.493533455	Lipidmaps	HMDB0112649	LMGP03010647
858.5260627	1-(7Z10Z13Z16Z-docosatetraenoyl)-2-(5Z8Z11Z14Z-eicosatetraenoyl)-glycero-3-phosphoserine	1-(7Z,10Z,13Z,16Z-docosatetraenoyl)-2-(5Z,8Z,11Z,14Z-eicosatetraenoyl)-glycero-3-phosphoserine	$C_{48}H_{78}NO_{10}P$	Phosphatidylserines	(1) Phosphatidylcholine Biosynthesis; (2)Phosphatidylethanolamine Biosynthesis	M-H	858.529062	3.493533455	Lipidmaps	HMDB0112805	LMGP03010810
858.5260627	1-(4Z7Z10Z13Z16Z19Z-docosahexaenoyl)-2-(11Z14Z-eicosadienoyl)-glycero-3-phosphoserine	1-(4Z,7Z,10Z,13Z,16Z,19Z-docosahexaenoyl)-2-(11Z,14Z-eicosadienoyl)-glycero-3-phosphoserine	$C_{48}H_{78}NO_{10}P$	Phosphatidylserines	(1) Phosphatidylcholine Biosynthesis; (2)Phosphatidylethanolamine Biosynthesis	M-H	858.529062	3.493533455	Lipidmaps	HMDB0112870	LMGP03010837
743.5455993	Unknown										
857.52607	Unknown										
740.5255212	PC(15:018:3(6Z9Z12Z))	1-Pentadecanoyl-2-g-linolenoyl-sn-glycero-3-phosphocholine	$C_{41}H_{76}NO_8P$	Phosphatidylcholines	(1) Phosphatidylcholine Biosynthesis; (2)Phosphatidylethanolamine Biosynthesis	M-H	740.5235789	2.622900952	HMDB	HMDB07941	HMDB07941
740.5255212	PC(15:018:3(9Z12Z15Z))	1-Pentadecanoyl-2-a-linolenoyl-sn-glycero-3-phosphocholine	$C_{41}H_{76}NO_8P$	Phosphatidylcholines	(1) Phosphatidylcholine Biosynthesis; (2)Phosphatidylethanolamine Biosynthesis	M-H	740.5235789	2.622900952	HMDB	HMDB07942	HMDB07942
740.5255212	PC(18:3(6Z9Z12Z)15:0)	1-g-Linolenoyl-2-pentadecanoyl-sn-glycero-3-phosphocholine	$C_{41}H_{76}NO_8P$	Phosphatidylcholines	(1) Phosphatidylcholine Biosynthesis; (2)Phosphatidylethanolamine Biosynthesis	M-H	740.5235789	2.622900952	HMDB	HMDB08165	HMDB08165
740.5255212	PC(18:3(9Z12Z15Z)15:0)	1-a-Linolenoyl-2-pentadecanoyl-sn-glycero-3-phosphocholine	$C_{41}H_{76}NO_8P$	Phosphatidylcholines	(1) Phosphatidylcholine Biosynthesis; (2)Phosphatidylethanolamine Biosynthesis	M-H	740.5235789	2.622900952	HMDB	HMDB08198	HMDB08198
740.5255212	PE(14:1(9Z)22:2(13Z16Z))	1-Myristoleoyl-2-docosadienoyl-sn-glycero-3-phosphoethanolamine	$C_{41}H_{76}NO_8P$	Phosphatidylethanolamines	(1) Phosphatidylcholine Biosynthesis; (2)Phosphatidylethanolamine Biosynthesis	M-H	740.5235789	2.622900952	HMDB	HMDB08876	HMDB08876
740.5255212	PE(16:020:3(5Z8Z11Z))	1-Palmitoyl-2-meadoyl-sn-glycero-3-phosphoethanolamine	$C_{41}H_{76}NO_8P$	Phosphatidylethanolamines	(1) Phosphatidylcholine Biosynthesis; (2)Phosphatidylethanolamine Biosynthesis	M-H	740.5235789	2.622900952	HMDB	HMDB08935	HMDB08935
740.5255212	PE(16:020:3(8Z11Z14Z))	1-Hexadecanoyl-2-(8Z,11Z,14Z-eicosatrienoyl)-glycero-3-phosphoethanolamine	$C_{41}H_{76}NO_8P$	Phosphatidylethanolamines	(1) Phosphatidylcholine Biosynthesis; (2)Phosphatidylethanolamine Biosynthesis	M-H	740.5235789	2.622900952	HMDB	HMDB08936	HMDB08936
740.5255212	PE(16:1(9Z)20:2(11Z14Z))	1-Palmitoleoyl-2-eicosadienoyl-sn-glycero-3-phosphoethanolamine	$C_{41}H_{76}NO_8P$	Phosphatidylethanolamines	(1) Phosphatidylcholine Biosynthesis; (2)Phosphatidylethanolamine Biosynthesis	M-H	740.5235789	2.622900952	HMDB	HMDB08967	HMDB08967
740.5255212	PE(18:018:3(6Z9Z12Z))	1-Stearoyl-2-a-linolenoyl-sn-glycero-3-phosphoethanolamine	$C_{41}H_{76}NO_8P$	Phosphatidylethanolamines	(1) Phosphatidylcholine Biosynthesis; (2)Phosphatidylethanolamine Biosynthesis	M-H	740.5235789	2.622900952	HMDB	HMDB08995	HMDB08995
740.5255212	PE(18:018:3(9Z12Z15Z))	1-Vaccenoyl-2-linoleoyl-sn-glycero-3-phosphoethanolamine	$C_{41}H_{76}NO_8P$	Phosphatidylethanolamines	(1) Phosphatidylcholine Biosynthesis; (2)Phosphatidylethanolamine Biosynthesis	M-H	740.5235789	2.622900952	HMDB	HMDB08996	HMDB08996
740.5255212	PE(18:1(11Z)18:2(9Z12Z))	1-(9Z)-Octadecenoyl-2-(9Z,12Z)-octadecadienoyl-sn-glycero-3-phosphoethanolamine	$C_{41}H_{76}NO_8P$	Phosphatidylethanolamines	(1) Phosphatidylcholine Biosynthesis; (2)Phosphatidylethanolamine Biosynthesis	M-H	740.5235789	2.622900952	HMDB	HMDB09027	HMDB09027
740.5255212	PE(18:1(9Z)18:2(9Z12Z))	1-(9Z)-Octadecenoyl-2-(9Z,12Z)-octadecadienoyl-sn-glycero-3-phosphoethanolamine	$C_{41}H_{76}NO_8P$	Phosphatidylethanolamines	(1) Phosphatidylcholine Biosynthesis; (2)Phosphatidylethanolamine Biosynthesis	M-H	740.5235789	2.622900952	HMDB	HMDB09060	HMDB09060
740.5255212	PE(18:2(9Z12Z)18:1(11Z))	1-Linoleoyl-2-vaccenoyl-sn-glycero-3-phosphoethanolamine	$C_{41}H_{76}NO_8P$	Phosphatidylethanolamines	(1) Phosphatidylcholine Biosynthesis; (2)Phosphatidylethanolamine Biosynthesis	M-H	740.5235789	2.622900952	HMDB	HMDB09091	HMDB09091
740.5255212	PE(18:2(9Z12Z)18:1(9Z))	1-Linoleoyl-2-oleoyl-sn-glycero-3-phosphoethanolamine	$C_{41}H_{76}NO_8P$	Phosphatidylethanolamines	(1) Phosphatidylcholine Biosynthesis; (2)Phosphatidylethanolamine Biosynthesis	M-H	740.5235789	2.622900952	HMDB	HMDB09092	HMDB09092
740.5255212	PE(18:3(6Z9Z12Z)18:0)	1-g-Linolenoyl-2-stearoyl-sn-glycero-3-phosphoethanolamine	$C_{41}H_{76}NO_8P$	Phosphatidylethanolamines	(1) Phosphatidylcholine Biosynthesis; (2)Phosphatidylethanolamine Biosynthesis	M-H	740.5235789	2.622900952	HMDB	HMDB09123	HMDB09123
740.5255212	PE(18:3(9Z12Z15Z)18:0)	1-alpha-Linolenoyl-2-stearoyl-sn-glycero-3-phosphoethanolamine	$C_{41}H_{76}NO_8P$	Phosphatidylethanolamines	(1) Phosphatidylcholine Biosynthesis; (2)Phosphatidylethanolamine Biosynthesis	M-H	740.5235789	2.622900952	HMDB	HMDB09156	HMDB09156
740.5255212	PE(20:2(11Z14Z)16:1(9Z))	1-Eicosadienoyl-2-palmitoleoyl-sn-glycero-3-phosphoethanolamine	$C_{41}H_{76}NO_8P$	Phosphatidylethanolamines	(1) Phosphatidylcholine Biosynthesis; (2)Phosphatidylethanolamine Biosynthesis	M-H	740.5235789	2.622900952	HMDB	HMDB09287	HMDB09287
740.5255212	PE(20:3(5Z8Z11Z)16:0)	1-Meadoyl-2-palmitoyl-sn-glycero-3-phosphoethanolamine	$C_{41}H_{76}NO_8P$	Phosphatidylethanolamines	(1) Phosphatidylcholine Biosynthesis; (2)Phosphatidylethanolamine Biosynthesis	M-H	740.5235789	2.622900952	HMDB	HMDB09319	HMDB09319
740.5255212	PE(20:3(8Z11Z14Z)16:0)	1-Homo-g-linolenoyl-2-palmitoyl-sn-glycero-3-phosphoethanolamine	$C_{41}H_{76}NO_8P$	Phosphatidylethanolamines	(1) Phosphatidylcholine Biosynthesis; (2)Phosphatidylethanolamine Biosynthesis	M-H	740.5235789	2.622900952	HMDB	HMDB09352	HMDB09352
740.5255212	PE(22:2(13Z16Z)14:1(9Z))	1-Docosadienoyl-2-myristoleoyl-sn-glycero-3-phosphoethanolamine	$C_{41}H_{76}NO_8P$	Phosphatidylethanolamines	(1) Phosphatidylcholine Biosynthesis; (2)Phosphatidylethanolamine Biosynthesis	M-H	740.5235789	2.622900952	HMDB	HMDB09548	HMDB09548
742.5356066	PC(15:018:2(9Z12Z))	1-Pentadecanoyl-2-(9Z,12Z-octadecadienoyl)-sn-glycero-3-phosphocholine	$C_{41}H_{78}NO_8P$	Phosphatidylcholines	(1) Phosphatidylcholine Biosynthesis; (2)Phosphatidylethanolamine Biosynthesis	M-H	742.539229	4.878395455	HMDB	HMDB07940	HMDB07940
742.5356066	PC(18:2(9Z12Z)15:0)	1-linoleoyl-2-pentadecanoyl-sn-glycero-3-phosphocholine	$C_{41}H_{78}NO_8P$	Phosphatidylcholines	(1) Phosphatidylcholine Biosynthesis; (2)Phosphatidylethanolamine Biosynthesis	M-H	742.539229	4.878395455	HMDB	HMDB08132	HMDB08132
742.5356066	PE(14:022:2(13Z16Z))	1-Myristoyl-2-docosadienoyl-sn-glycero-3-phosphoethanolamine	$C_{41}H_{78}NO_8P$	Phosphatidylethanolamines	(1) Phosphatidylcholine Biosynthesis; (2)Phosphatidylethanolamine Biosynthesis	M-H	742.539229	4.878395455	HMDB	HMDB08843	HMDB08843
742.5356066	PE(14:1(9Z)22:1(13Z))	1-Myristoleoyl-2-erucoyl-sn-glycero-3-phosphoethanolamine	$C_{41}H_{78}NO_8P$	Phosphatidylethanolamines	(1) Phosphatidylcholine Biosynthesis; (2)Phosphatidylethanolamine Biosynthesis	M-H	742.539229	4.878395455	HMDB	HMDB08875	HMDB08875
742.5356066	PE(16:020:2(11Z14Z))	1-g-Linolenoyl-2-vaccenoyl-sn-glycero-3-phosphoethanolamine	$C_{41}H_{78}NO_9P$	Phosphatidylethanolamines	(1) Phosphatidylcholine Biosynthesis; (3)Phosphatidylethanolamine Biosynthesis	M-H	742.539229	4.878395455	HMDB	HMDB08934	HMDB08934
742.5356066	PE(16:1(9Z)20:1(11Z))	1-Palmitoleoyl-2-eicosenoyl-sn-glycero-3-phosphoethanolamine	$C_{41}H_{78}NO_{10}P$	Phosphatidylethanolamines	(1) Phosphatidylcholine Biosynthesis; (4)Phosphatidylethanolamine Biosynthesis	M-H	742.539229	4.878395455	HMDB	HMDB08966	HMDB08966
742.5356066	PE(18:018:2(9Z12Z))	1,2-Divaccenoyl-rac-glycero-3-phosphoethanolamine	$C_{41}H_{78}NO_{11}P$	Phosphatidylethanolamines	(1) Phosphatidylcholine Biosynthesis; (5)Phosphatidylethanolamine Biosynthesis	M-H	742.539229	4.878395455	HMDB	HMDB08994	HMDB08994
742.5356066	PE(18:1(11Z)18:1(11Z))	1,2-Divaccenoyl-rac-glycero-3-phosphoethanolamine	$C_{41}H_{78}NO_{12}P$	Phosphatidylethanolamines	(1) Phosphatidylcholine Biosynthesis; (6)Phosphatidylethanolamine Biosynthesis	M-H	742.539229	4.878395455	HMDB	HMDB09025	HMDB09025
742.5356066	PE(18:1(11Z)18:1(9Z))	1-Vaccenoyl-2-oleoyl-sn-glycero-3-phosphoethanolamine	$C_{41}H_{78}NO_{13}P$	Phosphatidylethanolamines	(1) Phosphatidylcholine Biosynthesis; (7)Phosphatidylethanolamine Biosynthesis	M-H	742.539229	4.878395455	HMDB	HMDB09026	HMDB09026
742.5356066	PE(18:1(9Z)18:1(11Z))	1-Oleoyl-2-vaccenoyl-sn-glycero-3-phosphoethanolamine	$C_{41}H_{78}NO_{14}P$	Phosphatidylethanolamines	(1) Phosphatidylcholine Biosynthesis; (8)Phosphatidylethanolamine Biosynthesis	M-H	742.539229	4.878395455	HMDB	HMDB09058	HMDB09058
742.5356066	PE(18:1(9Z)18:1(9Z))	1,2-Di-(9Z-octadecenoyl)-sn-glycero-3-phosphoethanolamine	$C_{41}H_{78}NO_{15}P$	Phosphatidylethanolamines	(1) Phosphatidylcholine Biosynthesis; (9)Phosphatidylethanolamine Biosynthesis	M-H	742.539229	4.878395455	HMDB	HMDB09059	HMDB09059
742.5356066	PE(18:2(9Z12Z)18:0)	1-Linoleoyl-2-stearoyl-sn-glycero-3-phosphoethanolamine	$C_{41}H_{78}NO_{16}P$	Phosphatidylethanolamines	(1) Phosphatidylcholine Biosynthesis; (10)Phosphatidylethanolamine Biosynthesis	M-H	742.539229	4.878395455	HMDB	HMDB09090	HMDB09090
742.5356066	PE(20:1(11Z)16:1(9Z))	1-Eicosenoyl-2-palmitoleoyl-sn-glycero-3-phosphoethanolamine	$C_{41}H_{78}NO_{17}P$	Phosphatidylethanolamines	(1) Phosphatidylcholine Biosynthesis; (11)Phosphatidylethanolamine Biosynthesis	M-H	742.539229	4.878395455	HMDB	HMDB09254	HMDB09254
742.5356066	PE(20:2(11Z14Z)16:0)	1-Eicosadienoyl-2-palmitoyl-sn-glycero-3-phosphoethanolamine	$C_{41}H_{78}NO_{18}P$	Phosphatidylethanolamines	(1) Phosphatidylcholine Biosynthesis; (12)Phosphatidylethanolamine Biosynthesis	M-H	742.539229	4.878395455	HMDB	HMDB09286	HMDB09286
742.5356066	PE(22:1(13Z)14:1(9Z))	1-Erucoyl-2-myristoleoyl-sn-glycero-3-phosphoethanolamine	$C_{41}H_{78}NO_{19}P$	Phosphatidylethanolamines	(1) Phosphatidylcholine Biosynthesis; (13)Phosphatidylethanolamine Biosynthesis	M-H	742.539229	4.878395455	HMDB	HMDB09515	HMDB09515
742.5356066	PE(22:2(13Z16Z)14:0)	1-Docosadienoyl-2-myristoyl-sn-glycero-3-phosphoethanolamine	$C_{41}H_{78}NO_{20}P$	Phosphatidylethanolamines	(1) Phosphatidylcholine Biosynthesis; (14)Phosphatidylethanolamine Biosynthesis	M-H	742.539229	4.878395455	HMDB	HMDB09547	HMDB09547
280.2335701	Unknown										
861.5461407	PI(16:020:2(11Z14Z))	1-Palmitoyl-2-eicosadienoyl-sn-glycero-3-phosphoinositol	$C_{45}H_{83}O_{13}P$	Phosphatidylinositols	NA	M-H	861.5498533	4.309176057	HMDB	HMDB09786	HMDB09786
861.5461407	PI(18:018:2(9Z12Z))	1-Stearoyl-2-linoleoyl-sn-glycero-3-phosphoinositol	$C_{45}H_{83}O_{13}P$	Phosphatidylinositols	NA	M-H	861.5498533	4.309176057	HMDB	HMDB09809	HMDB09809
861.5461407	PI(18:1(11Z)18:1(11Z))	1,2-Divaccenoyl-rac-glycero-3-phosphoinositol	$C_{45}H_{83}O_{13}P$	Phosphatidylinositols	NA	M-H	861.5498533	4.309176057	HMDB	HMDB09824	HMDB09824
861.5461407	PI(18:1(11Z)18:1(9Z))	1-vaccenoyl-2-oleoyl-sn-glycero-3-phosphoinositol	$C_{45}H_{83}O_{13}P$	Phosphatidylinositols	NA	M-H	861.5498533	4.309176057	HMDB	HMDB09825	HMDB09825
861.5461407	PI(18:1(9Z)18:1(11Z))	1-Oleoyl-2-vaccenoyl-sn-glycero-3-phosphoinositol	$C_{45}H_{83}O_{13}P$	Phosphatidylinositols	NA	M-H	861.5498533	4.309176057	HMDB	HMDB09836	HMDB09836
861.5461407	PI(18:1(9Z)18:1(9Z))	1,2-Dioleoyl-rac-glycero-3-phosphoinositol	$C_{45}H_{83}O_{13}P$	Phosphatidylinositols	NA	M-H	861.5498533	4.309176057	HMDB	HMDB09837	HMDB09837
861.5461407	PI(18:2(9Z12Z)18:0)	1-Linoleoyl-2-stearoyl-sn-glycero-3-phosphoinositol	$C_{45}H_{83}O_{13}P$	Phosphatidylinositols	NA	M-H	861.5498533	4.309176057	HMDB	HMDB09847	HMDB09847
861.5461407	PI(20:2(11Z14Z)16:0)	1-Eicosadienoyl-2-palmitoyl-sn-glycero-3-phosphoinositol	$C_{45}H_{83}O_{13}P$	Phosphatidylinositols	NA	M-H	861.5498533	4.309176057	HMDB	HMDB09875	HMDB09875

Table 2: **Identified potential biomarkers of colorectal adenocarcinoma**

m/z	compound	formula	structural formula	class	pathway	adduct	benchmark m/z	ppm difference	database	HMDB-ID	database-ID
279.2335774	Linoleic acid	(9Z,12Z)-Octadecadienoic acid	$C_{18}H_{32}O_2$	Long-chain fatty acids	NA	M-H	279.2329543	2.231470141	HMDB	HMDB00673	HMDB00673
279.2335774	Bovinic acid	(9Z,11E)-Octadecadienoic acid	$C_{18}H_{32}O_2$	Long-chain fatty acids	NA	M-H	279.2329543	2.231470141	HMDB	HMDB03797	HMDB03797
279.2335774	9E11E-Octadecadienoic acid	(9E,11E)-Octadecadienoic acid	$C_{18}H_{32}O_2$	Long-chain fatty acids	NA	M-H	279.2329543	2.231470141	HMDB	HMDB05047	HMDB05047
279.2335774	10E12Z-Octadecadienoic acid	(10E,12Z)-Octadecadienoic acid	$C_{18}H_{32}O_2$	Long-chain fatty acids	NA	M-H	279.2329543	2.231470141	HMDB	HMDB05048	HMDB05048
279.2335774	Linoelaidic acid	Linoelaidic acid	$C_{18}H_{32}O_2$	Long-chain fatty acids	NA	M-H	279.2329543	2.231470141	HMDB	HMDB06270	HMDB06270
279.2335774	3-Oxooctadecanoic acid	3-Oxooctadecanoic acid	C18H34O3	Long-chain fatty acids	NA	M-H2O-H	279.232405	4.19865309	HMDB	HMDB10736	HMDB10736
279.2335774	Mangiferic acid	9,15-Octadecadienoic acid	$C_{18}H_{32}O_2$	Long-chain fatty acids	NA	M-H	279.2329543	2.231470141	HMDB	HMDB29800	HMDB29800
279.2335774	Linalyl caprylate	1-Ethenyl-1,5-dimethyl-4-hexenyl octanoate	$C_{18}H_{32}O_2$	Long-chain fatty acids	NA	M-H	279.2329543	2.231470141	HMDB	HMDB30430	HMDB30430
279.2335774	9-Oxooctadecanoic acid	9-Oxooctadecanoic acid	$C_{18}H_{32}O_2$	Long-chain fatty acids	NA	M-H2O-H	279.232405	4.19865309	HMDB	HMDB30979	HMDB30979
279.2335774	10-Oxooctadecanoic acid	10-Oxooctadecanoic acid	$C_{18}H_{32}O_2$	Long-chain fatty acids	NA	M-H2O-H	279.232405	4.19865309	HMDB	HMDB30980	HMDB30980
279.2335774	11-Oxooctadecanoic acid	11-Oxooctadecanoic acid	$C_{18}H_{32}O_2$	Long-chain fatty acids	NA	M-H2O-H	279.232405	4.19865309	HMDB	HMDB30981	HMDB30981
279.2335774	Ethyl 2E4Z-hexadecadienoate	Ethyl 2E,4Z-hexadecadienoic acid	$C_{18}H_{32}O_2$	Long-chain fatty acids	NA	M-H	279.2329543	2.231470141	HMDB	HMDB31051	HMDB31051
279.2335774	5-Octadecynoic acid	5-Octadecynoic acid	$C_{18}H_{32}O_2$	Long-chain fatty acids	NA	M-H	279.2329543	2.231470141	HMDB	HMDB31097	HMDB31097
279.2335774	5-Hexyltetrahydro-2-furanoctanoic acid	9,12-Epoxyoctadecanoic acid	$C_{18}H_{32}O_2$	Long-chain fatty acids	NA	M-H2O-H	279.232405	4.19865309	HMDB	HMDB31127	HMDB31127
279.2335774	5-Oxooctadecanoic acid	5-Oxooctadecanoic acid	$C_{18}H_{32}O_2$	Long-chain fatty acids	NA	M-H2O-H	279.232405	4.19865309	HMDB	HMDB34074	HMDB34074
279.2335774	Ricinoleic acid	12-Hydroxyoleic acid	$C_{18}H_{32}O_2$	Long-chain fatty acids	NA	M-H2O-H	279.232405	4.19865309	HMDB	HMDB34297	HMDB34297
717.5254886	DG(18:2n60:022:4n6)	1-Linoleoyl-3-adrenoyl-sn-glycerol	$C_{48}H_{87}O_{10}P$	Diradylglycerols	NA	M+Cl	717.5230275	3.4299387	HMDB	HMDB56288	HMDB56288
717.5254886	DG(20:2n60:022:4n6)	1-Eicosadienoyl-3-adrenoyl-sn-glycerol	$C_{48}H_{87}O_{10}P$	Diradylglycerols	NA	M+Cl	717.5230275	3.4299387	HMDB	HMDB56313	HMDB56313
717.5254886	1-nonadecanoyl-2-(4Z7Z10Z13Z16Z19Z-docosahexaenoyl)-sn-glycerol	1-nonadecanoyl-2-(4Z,7Z,10Z,13Z,16Z,19Z-docosahexaenoyl)-sn-glycerol	$C_{48}H_{87}O_{10}P$	Diradylglycerols	NA	M+Cl	717.523028	3.429241856	Lipidmaps	NA	LMGL02010250
717.5254886	1-9Z-nonadecenoyl-2-(7Z10Z13Z16Z19Z-docosapentaenoyl)-sn-glycerol	1-9Z-nonadecenoyl-2-(7Z,10Z,13Z,16Z,19Z-docosapentaenoyl)-sn-glycerol	$C_{48}H_{87}O_{10}P$	Diradylglycerols	NA	M+Cl	717.523028	3.429241856	Lipidmaps	NA	LMGL02010544
717.5254886	1-eicosyl-2-(5Z8Z11Z14Z17Z-eicosapentaenoyl)-glycero-3-phosphate	1-eicosyl-2-(5Z,8Z,11Z,14Z,17Z-eicosapentaenoyl)-glycero-3-phosphate	$C_{48}H_{87}O_{10}P$	Diradylglycerols	NA	M-H2O-H	717.522304	4.438273183	Lipidmaps	NA	LMGP10020065
717.5254886	1-(1Z-octadecenyl)-2-(7Z10Z13Z16Z-docosatetraenoyl)-glycero-3-phosphate	1-(1Z-octadecenyl)-2-(7Z,10Z,13Z,16Z-docosatetraenoyl)-glycero-3-phosphate	$C_{48}H_{87}O_{10}P$	Diradylglycerols	NA	M-H2O-H	717.522304	4.438273183	Lipidmaps	NA	LMGP10030055
717.5254886	1-(1Z-eicosenyl)-2-(5Z8Z11Z14Z-eicosatetraenoyl)-glycero-3-phosphate	1-(1Z-eicosenyl)-2-(5Z,8Z,11Z,14Z-eicosatetraenoyl)-glycero-3-phosphate	$C_{48}H_{87}O_{10}P$	Diradylglycerols	NA	M-H2O-H	717.522304	4.438273183	Lipidmaps	NA	LMGP10030079
889.5761368	1-eicosanoyl-2-(7Z10Z13Z16Z-docosatetraenoyl)-glycero-3-phospho-(1-sn-glycerol)	1-eicosanoyl-2-(7Z,10Z,13Z,16Z-docosatetraenoyl)-glycero-3-phospho-(1'-sn-glycerol)	$C_{48}H_{87}O_{10}P$	Diradylglycerols	NA	M+Cl	889.573091	3.423878297	Lipidmaps	NA	LMGP04010527
889.5761368	1-(11Z14Z-eicosadienoyl)-2-(13Z16Z-docosadienoyl)-glycero-3-phospho-(1-sn-glycerol)	1-(11Z,14Z-eicosadienoyl)-2-(13Z,16Z-docosadienoyl)-glycero-3-phospho-(1'-sn-glycerol)	$C_{48}H_{87}O_{10}P$	Diradylglycerols	NA	M+Cl	889.573091	3.423878297	Lipidmaps	NA	LMGP04010586
889.5761368	1-(8Z11Z14Z-eicosatrienoyl)-2-(11Z-docosenoyl)-glycero-3-phospho-(1-sn-glycerol)	1-(8Z,11Z,14Z-eicosatrienoyl)-2-(11Z-docosenoyl)-glycero-3-phospho-(1'-sn-glycerol)	$C_{48}H_{87}O_{10}P$	Diradylglycerols	NA	M+Cl	889.573091	3.423878297	Lipidmaps	NA	LMGP04010616
889.5761368	1-(5Z8Z11Z14Z-eicosatetraenoyl)-2-docosanoyl-glycero-3-phospho-(1-sn-glycerol)	1-(5Z,8Z,11Z,14Z-eicosatetraenoyl)-2-docosanoyl-glycero-3-phospho-(1'-sn-glycerol)	$C_{48}H_{87}O_{10}P$	Diradylglycerols	NA	M+Cl	889.573091	3.423878297	Lipidmaps	NA	LMGP04010644
889.5761368	1-docosanoyl-2-(5Z8Z11Z14Z-eicosatetraenoyl)-glycero-3-phospho-(1-sn-glycerol)	1-docosanoyl-2-(5Z,8Z,11Z,14Z-eicosatetraenoyl)-glycero-3-phospho-(1'-sn-glycerol)	$C_{48}H_{87}O_{10}P$	Diradylglycerols	NA	M+Cl	889.573091	3.423878297	Lipidmaps	NA	LMGP04010719
889.5761368	1-(11Z-docosenoyl)-2-(8Z11Z14Z-eicosatrienoyl)-glycero-3-phospho-(1-sn-glycerol)	1-(11Z-docosenoyl)-2-(8Z,11Z,14Z-eicosatrienoyl)-glycero-3-phospho-(1'-sn-glycerol)	$C_{48}H_{87}O_{10}P$	Diradylglycerols	NA	M+Cl	889.573091	3.423878297	Lipidmaps	NA	LMGP04010747
889.5761368	1-(13Z16Z-docosadienoyl)-2-(11Z14Z-eicosadienoyl)-glycero-3-phospho-(1-sn-glycerol)	1-(13Z,16Z-docosadienoyl)-2-(11Z,14Z-eicosadienoyl)-glycero-3-phospho-(1'-sn-glycerol)	$C_{48}H_{87}O_{10}P$	Diradylglycerols	NA	M+Cl	889.573091	3.423878297	Lipidmaps	NA	LMGP04010777
889.5761368	1-(7Z10Z13Z16Z-docosatetraenoyl)-2-eicosanoyl-glycero-3-phospho-(1-sn-glycerol)	1-(7Z,10Z,13Z,16Z-docosatetraenoyl)-2-eicosanoyl-glycero-3-phospho-(1'-sn-glycerol)	$C_{48}H_{87}O_{10}P$	Diradylglycerols	NA	M+Cl	889.573091	3.423878297	Lipidmaps	NA	LMGP04010806
720.495467	PC(15:018:4(6Z9Z12Z15Z))	1-Pentadecanoyl-2-stearidonoyl-sn-glycero-3-phosphocholine	$C_{41}H_{74}NO_8P$	Phosphatidylcholines	(1) Phosphatidylcholine Biosynthesis; (2)Phosphatidylethanolamine Biosynthesis	M-H2O-H	720.4968149	1.870861844	HMDB	HMDB07943	HMDB07943
720.495467	PC(18:4(6Z9Z12Z15Z)15:0)	1-Stearidonoyl-2-pentadecanoyl-sn-glycero-3-phosphocholine	$C_{41}H_{74}NO_8P$	Phosphatidylcholines	(1) Phosphatidylcholine Biosynthesis; (2)Phosphatidylethanolamine Biosynthesis	M-H2O-H	720.4968149	1.870861844	HMDB	HMDB08231	HMDB08231
720.495467	PE(14:022:4(7Z10Z13Z16Z))	1-Myristoyl-2-adrenoyl-sn-glycero-3-phosphoethanolamine	$C_{41}H_{74}NO_8P$	Phosphatidylethanolamines	(1) Phosphatidylcholine Biosynthesis; (2)Phosphatidylethanolamine Biosynthesis	M-H2O-H	720.4968149	1.870861844	HMDB	HMDB08844	HMDB08844
720.495467	PE(16:020:4(5Z8Z11Z14Z))	1-Hexadecanoyl-2-(5Z,8Z,11Z,14Z-eicosatetraenoyl)-sn-glycero-3-phosphoethanolamine	$C_{41}H_{74}NO_8P$	Phosphatidylethanolamines	(1) Phosphatidylcholine Biosynthesis; (2)Phosphatidylethanolamine Biosynthesis	M-H2O-H	720.4968149	1.870861844	HMDB	HMDB08937	HMDB08937
720.495467	PE(16:020:4(8Z11Z14Z17Z))	1-Palmitoyl-2-eicsoatetraenoyl-sn-glycero-3-phosphoethanolamine	$C_{41}H_{74}NO_8P$	Phosphatidylethanolamines	(1) Phosphatidylcholine Biosynthesis; (2)Phosphatidylethanolamine Biosynthesis	M-H2O-H	720.4968149	1.870861844	HMDB	HMDB08938	HMDB08938
720.495467	PE(16:1(9Z)20:3(5Z8Z11Z))	1-Palmitoleoyl-2-meadoyl-sn-glycero-3-phosphoethanolamine	$C_{41}H_{74}NO_8P$	Phosphatidylethanolamines	(1) Phosphatidylcholine Biosynthesis; (2)Phosphatidylethanolamine Biosynthesis	M-H2O-H	720.4968149	1.870861844	HMDB	HMDB08968	HMDB08968
720.495467	PE(18:018:4(6Z9Z12Z15Z))	1-Stearoyl-2-stearidonoyl-sn-glycero-3-phosphoethanolamine	$C_{41}H_{74}NO_8P$	Phosphatidylethanolamines	(1) Phosphatidylcholine Biosynthesis; (2)Phosphatidylethanolamine Biosynthesis	M-H2O-H	720.4968149	1.870861844	HMDB	HMDB08997	HMDB08997
720.495467	PE(18:1(11Z)18:3(6Z9Z12Z))	1-Vaccenoyl-2-g-linolenoyl-sn-glycero-3-phosphoethanolamine	$C_{41}H_{74}NO_8P$	Phosphatidylethanolamines	(1) Phosphatidylcholine Biosynthesis; (2)Phosphatidylethanolamine Biosynthesis	M-H2O-H	720.4968149	1.870861844	HMDB	HMDB09028	HMDB09028
720.495467	PE(18:1(11Z)18:3(9Z12Z15Z))	1-Vaccenoyl-2-a-linolenoyl-sn-glycero-3-phosphoethanolamine	$C_{41}H_{74}NO_8P$	Phosphatidylethanolamines	(1) Phosphatidylcholine Biosynthesis; (2)Phosphatidylethanolamine Biosynthesis	M-H2O-H	720.4968149	1.870861844	HMDB	HMDB09029	HMDB09029
720.495467	PE(18:1(9Z)18:3(6Z9Z12Z))	1-Oleoyl-2-g-linolenoyl-sn-glycero-3-phosphoethanolamine	$C_{41}H_{74}NO_8P$	Phosphatidylethanolamines	(1) Phosphatidylcholine Biosynthesis; (2)Phosphatidylethanolamine Biosynthesis	M-H2O-H	720.4968149	1.870861844	HMDB	HMDB09061	HMDB09061
720.495467	PE(18:1(9Z)18:3(9Z12Z15Z))	1-Oleoyl-2-a-linolenoyl-sn-glycero-3-phosphoethanolamine	$C_{41}H_{74}NO_8P$	Phosphatidylethanolamines	(1) Phosphatidylcholine Biosynthesis; (2)Phosphatidylethanolamine Biosynthesis	M-H2O-H	720.4968149	1.870861844	HMDB	HMDB09062	HMDB09062
720.495467	PE(18:2(9Z12Z)18:2(9Z12Z))	1,2-Di-linoleoyl-sn-glycero-3-phosphoethanolamine	$C_{41}H_{74}NO_8P$	Phosphatidylethanolamines	(1) Phosphatidylcholine Biosynthesis; (2)Phosphatidylethanolamine Biosynthesis	M-H2O-H	720.4968149	1.870861844	HMDB	HMDB09093	HMDB09093
720.495467	PE(18:3(6Z9Z12Z)18:1(11Z))	1-g-Linolenoyl-2-vaccenoyl-sn-glycero-3-phosphoethanolamine	$C_{41}H_{74}NO_8P$	Phosphatidylethanolamines	(1) Phosphatidylcholine Biosynthesis; (2)Phosphatidylethanolamine Biosynthesis	M-H2O-H	720.4968149	1.870861844	HMDB	HMDB09124	HMDB09124
720.495467	PE(18:3(6Z9Z12Z)18:1(9Z))	1-g-Linolenoyl-2-oleoyl-sn-glycero-3-phosphoethanolamine	$C_{41}H_{74}NO_8P$	Phosphatidylethanolamines	(1) Phosphatidylcholine Biosynthesis; (2)Phosphatidylethanolamine Biosynthesis	M-H2O-H	720.4968149	1.870861844	HMDB	HMDB09125	HMDB09125
720.495467	PE(18:3(9Z12Z15Z)18:1(11Z))	1-a-Linolenoyl-2-vaccenoyl-sn-glycero-3-phosphoethanolamine	$C_{41}H_{74}NO_8P$	Phosphatidylethanolamines	(1) Phosphatidylcholine Biosynthesis; (2)Phosphatidylethanolamine Biosynthesis	M-H2O-H	720.4968149	1.870861844	HMDB	HMDB09157	HMDB09157
720.495467	PE(18:3(9Z12Z15Z)18:1(9Z))	1-a-Linolenoyl-2-oleoyl-sn-glycero-3-phosphoethanolamine	$C_{41}H_{74}NO_8P$	Phosphatidylethanolamines	(1) Phosphatidylcholine Biosynthesis; (2)Phosphatidylethanolamine Biosynthesis	M-H2O-H	720.4968149	1.870861844	HMDB	HMDB09158	HMDB09158
720.495467	PE(18:4(6Z9Z12Z15Z)18:0)	1-Stearidonoyl-2-stearoyl-sn-glycero-3-phosphoethanolamine	$C_{41}H_{74}NO_8P$	Phosphatidylethanolamines	(1) Phosphatidylcholine Biosynthesis; (2)Phosphatidylethanolamine Biosynthesis	M-H2O-H	720.4968149	1.870861844	HMDB	HMDB09189	HMDB09189
720.495467	PE(18:4(6Z9Z12Z15Z)P-18:1(11Z))	1-(6Z,9Z,12Z,15Z-Octadecatetraenoyl)-2-(1Z,11Z-octadecadienyl)-sn-glycero-3-phosphoethanolamine	$C_{41}H_{74}NO_8P$	Phosphatidylethanolamines	(1) Phosphatidylcholine Biosynthesis; (2)Phosphatidylethanolamine Biosynthesis	M-H	720.4973642	2.633250438	HMDB	HMDB09215	HMDB09215
720.495467	PE(18:4(6Z9Z12Z15Z)P-18:1(9Z))	1-(6Z,9Z,12Z,15Z-Octadecatetraenoyl)-2-(1Z,9Z-octadecadienyl)-sn-glycero-3-phosphoethanolamine	$C_{41}H_{74}NO_8P$	Phosphatidylethanolamines	(1) Phosphatidylcholine Biosynthesis; (2)Phosphatidylethanolamine Biosynthesis	M-H	720.4973642	2.633250438	HMDB	HMDB09216	HMDB09216
720.495467	PE(20:3(5Z8Z11Z)16:1(9Z))	1-Meadoyl-2-palmitoleoyl-sn-glycero-3-phosphoethanolamine	$C_{41}H_{74}NO_8P$	Phosphatidylethanolamines	(1) Phosphatidylcholine Biosynthesis; (2)Phosphatidylethanolamine Biosynthesis	M-H2O-H	720.4968149	1.870861844	HMDB	HMDB09320	HMDB09320
720.495467	PE(20:3(8Z11Z14Z)16:1(9Z))	1-Homo-g-linolenoyl-2-palmitoleoyl-sn-glycero-3-phosphoethanolamine	$C_{41}H_{74}NO_8P$	Phosphatidylethanolamines	(1) Phosphatidylcholine Biosynthesis; (2)Phosphatidylethanolamine Biosynthesis	M-H2O-H	720.4968149	1.870861844	HMDB	HMDB09353	HMDB09353
720.495467	PE(20:4(5Z8Z11Z14Z)16:0)	1-Arachidonoyl-2-palmitoyl-sn-glycero-3-phosphoethanolamine	$C_{41}H_{74}NO_8P$	Phosphatidylethanolamines	(1) Phosphatidylcholine Biosynthesis; (2)Phosphatidylethanolamine Biosynthesis	M-H2O-H	720.4968149	1.870861844	HMDB	HMDB09385	HMDB09385
720.495467	PE(20:4(8Z11Z14Z17Z)16:0)	1-Eicsoatetraenoyl-2-palmitoyl-sn-glycero-3-phosphoethanolamine	$C_{41}H_{74}NO_8P$	Phosphatidylethanolamines	(1) Phosphatidylcholine Biosynthesis; (2)Phosphatidylethanolamine Biosynthesis	M-H2O-H	720.4968149	1.870861844	HMDB	HMDB09418	HMDB09418
720.495467	PE(20:5(5Z8Z11Z14Z17Z)P-16:0)	1-(5Z,8Z,11Z,14Z,17Z-Eicosapentaenoyl)-2-(1Z-hexadecenyl)-sn-glycero-3-phosphoethanolamine	$C_{41}H_{74}NO_8P$	Phosphatidylethanolamines	(1) Phosphatidylcholine Biosynthesis; (2)Phosphatidylethanolamine Biosynthesis	M-H	720.4973642	2.633250438	HMDB	HMDB09477	HMDB09477
720.495467	PE(22:4(7Z10Z13Z16Z)14:0)	1-Adrenoyl-2-myristoyl-sn-glycero-3-phosphoethanolamine	$C_{41}H_{74}NO_8P$	Phosphatidylethanolamines	(1) Phosphatidylcholine Biosynthesis; (2)Phosphatidylethanolamine Biosynthesis	M-H2O-H	720.4968149	1.870861844	HMDB	HMDB09580	HMDB09580
720.495467	PE(P-16:020:5(5Z8Z11Z14Z17Z))	1-(1-Enyl-palmitoyl)-2-eicosapentaenoyl-sn-glycero-3-phosphoethanolamine	$C_{41}H_{74}NO_8P$	Phosphatidylethanolamines	(1) Phosphatidylcholine Biosynthesis; (2)Phosphatidylethanolamine Biosynthesis	M-H	720.4973642	2.633250438	HMDB	HMDB11354	HMDB11354
720.495467	PE(P-18:1(11Z)18:4(6Z9Z12Z15Z))	1-(1-Enyl-vaccenoyl)-2-stearidonoyl-sn-glycero-3-phosphoethanolamine	$C_{41}H_{74}NO_8P$	Phosphatidylethanolamines	(1) Phosphatidylcholine Biosynthesis; (2)Phosphatidylethanolamine Biosynthesis	M-H	720.4973642	2.633250438	HMDB	HMDB11412	HMDB11412
720.495467	PE(P-18:1(9Z)18:4(6Z9Z12Z15Z))	1-(1-Enyl-oleoyl)-2-stearidonoyl-sn-glycero-3-phosphoethanolamine	$C_{41}H_{74}NO_8P$	Phosphatidylethanolamines	(1) Phosphatidylcholine Biosynthesis; (2)Phosphatidylethanolamine Biosynthesis	M-H	720.4973642	2.633250438	HMDB	HMDB11445	HMDB11445
724.5155377	Unknown										
888.5662441	Unknown										
773.4356818	1-(1Z-hexadecenyl)-2-dodecanoyl-glycero-3-phospho-(1-myo-inositol)	1-(1Z-hexadecenyl)-2-dodecanoyl-glycero-3-phospho-(1'-myo-inositol)	$C_{32}H_{71}O_{12}P$	Phosphatidylinositols	NA	M+Cl	773.437721	2.636566519	Lipidmaps	NA	LMGP06030002

Table 3: **Indentified potential biomarkers of colorectal adenocarcinoma (Continuation)**. 14 m/z values are discovered to be strongly correlated with colorectal carcinoma. As checked with the Human Metabolome Database (HMDB), the LIPID MAPS® Structure Database (LMSD) and the MetaboAnalyst database, 9 out of 14 are successfully identified. For instance, m/z 858.5260627 is identified as compounds belonging to the class of phosphatidylserines with a tolerance window of 5 ppm. These compounds are speculated to be related to the vigorous phospholipid metabolism of colorectal cancer cells. Nevertheless, there is no relevant report on phosphatidylserine compounds as markers for colorectal cancer.

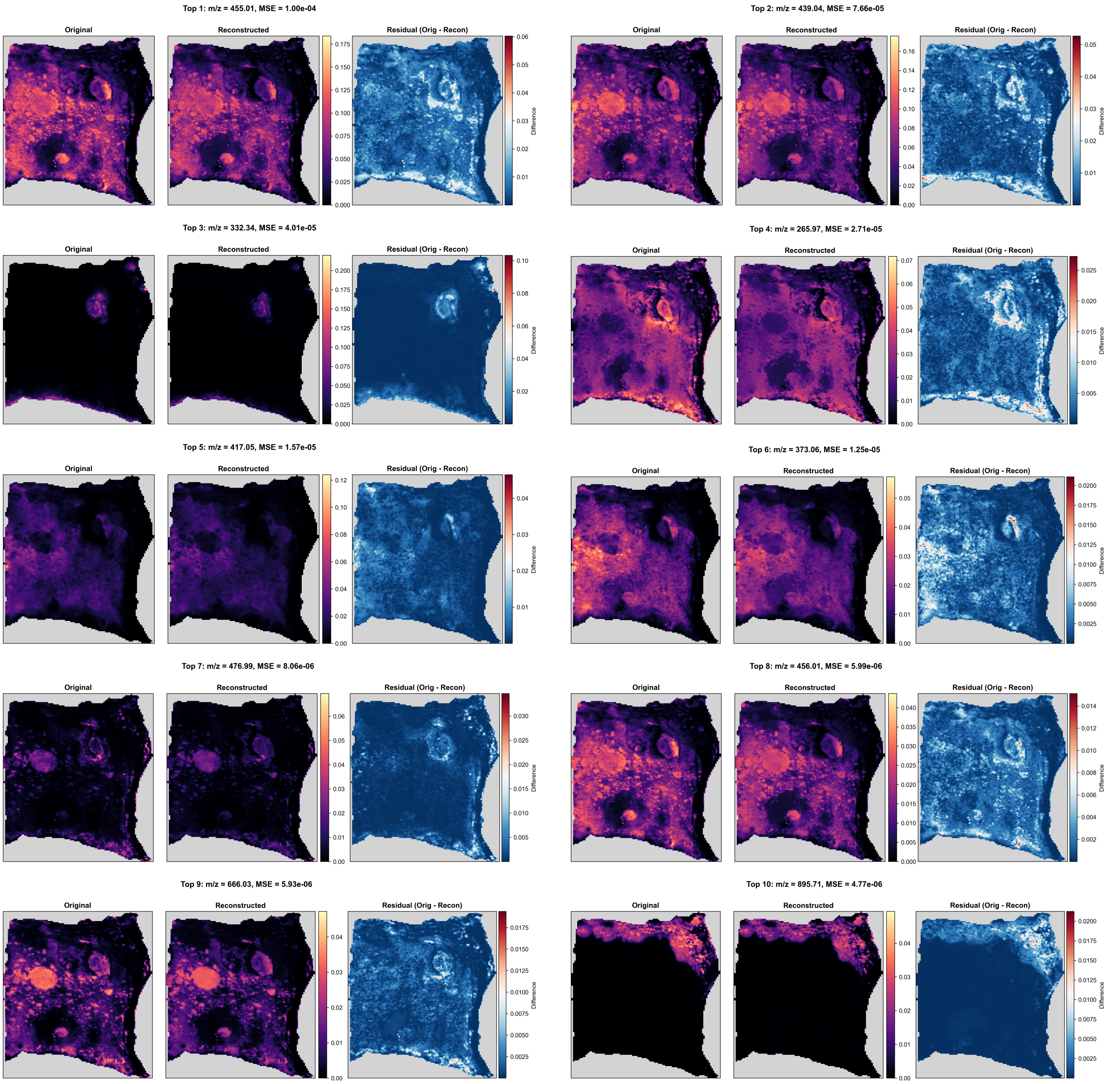

Figure 2: **Visualization of the m/z ions with Top 10 largest reconstruction error in the prostate dataset.**. Each visualization consists of four columns: the original spectrum, reconstructed spectrum, and residual error (absolute value).

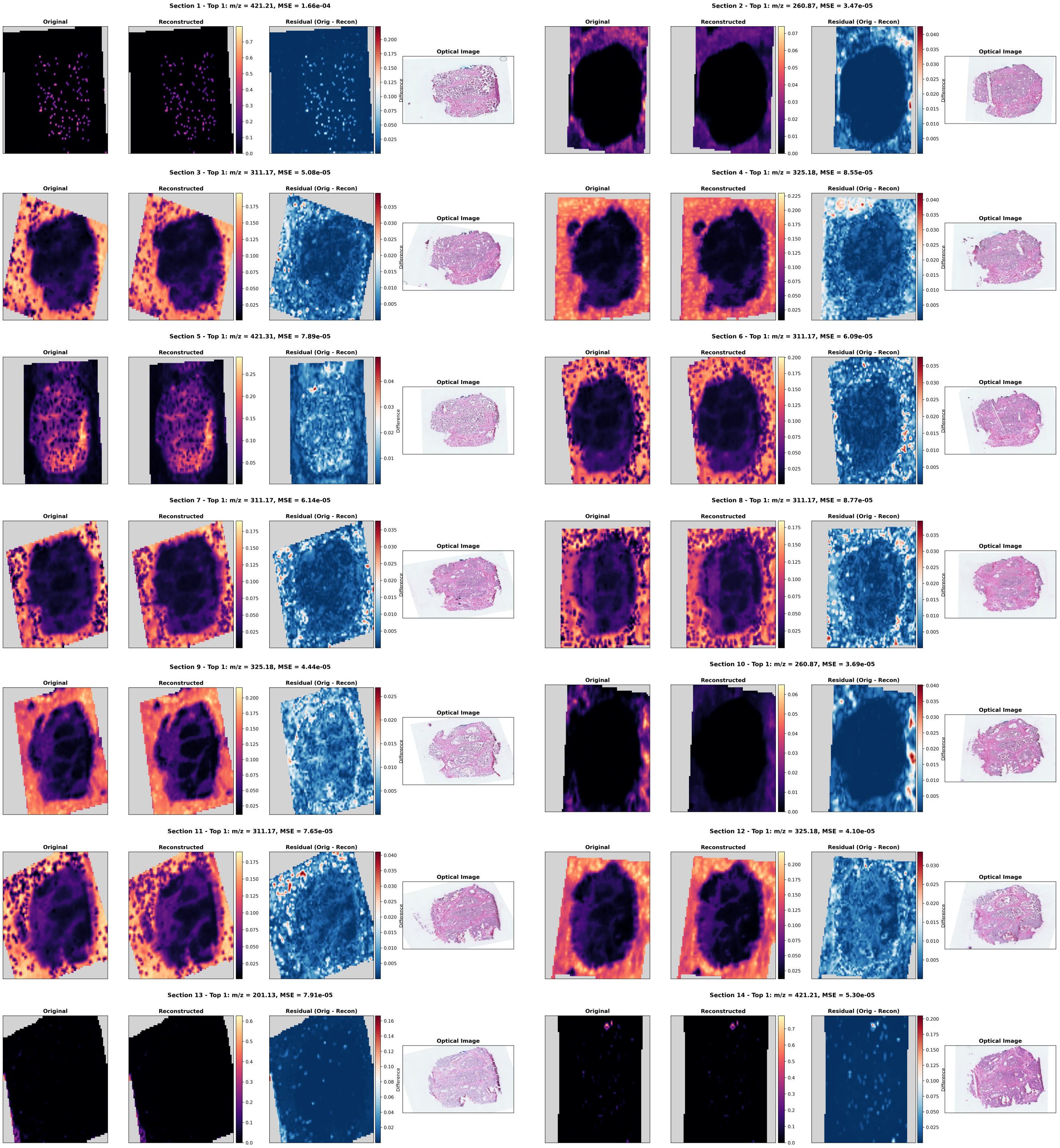

Figure 3: **Visualization of the m/z ion with the largest reconstruction error in each colorectal adenocarcinoma tissue section.(Section 1 to Section 14)**. Each visualization consists of four columns: the original spectrum, reconstructed spectrum, residual error (absolute value), and the optical pathology image.

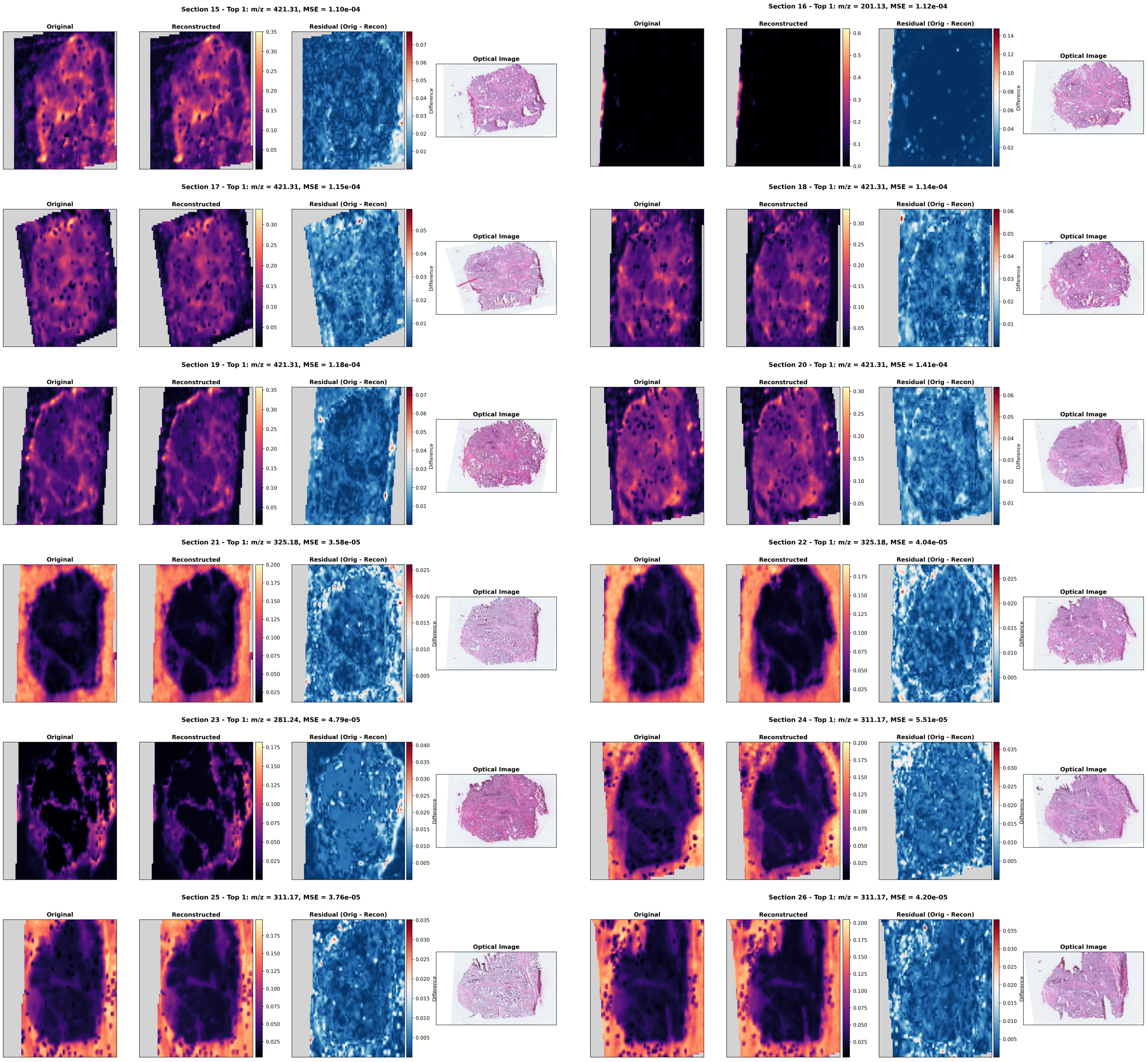

Figure 4: **Visualization of the m/z ion with the largest reconstruction error in each colorectal adenocarcinoma tissue section.(Section 15 to Section 26)**. Each visualization consists of four columns: the original spectrum, reconstructed spectrum, residual error (absolute value), and the optical pathology image.

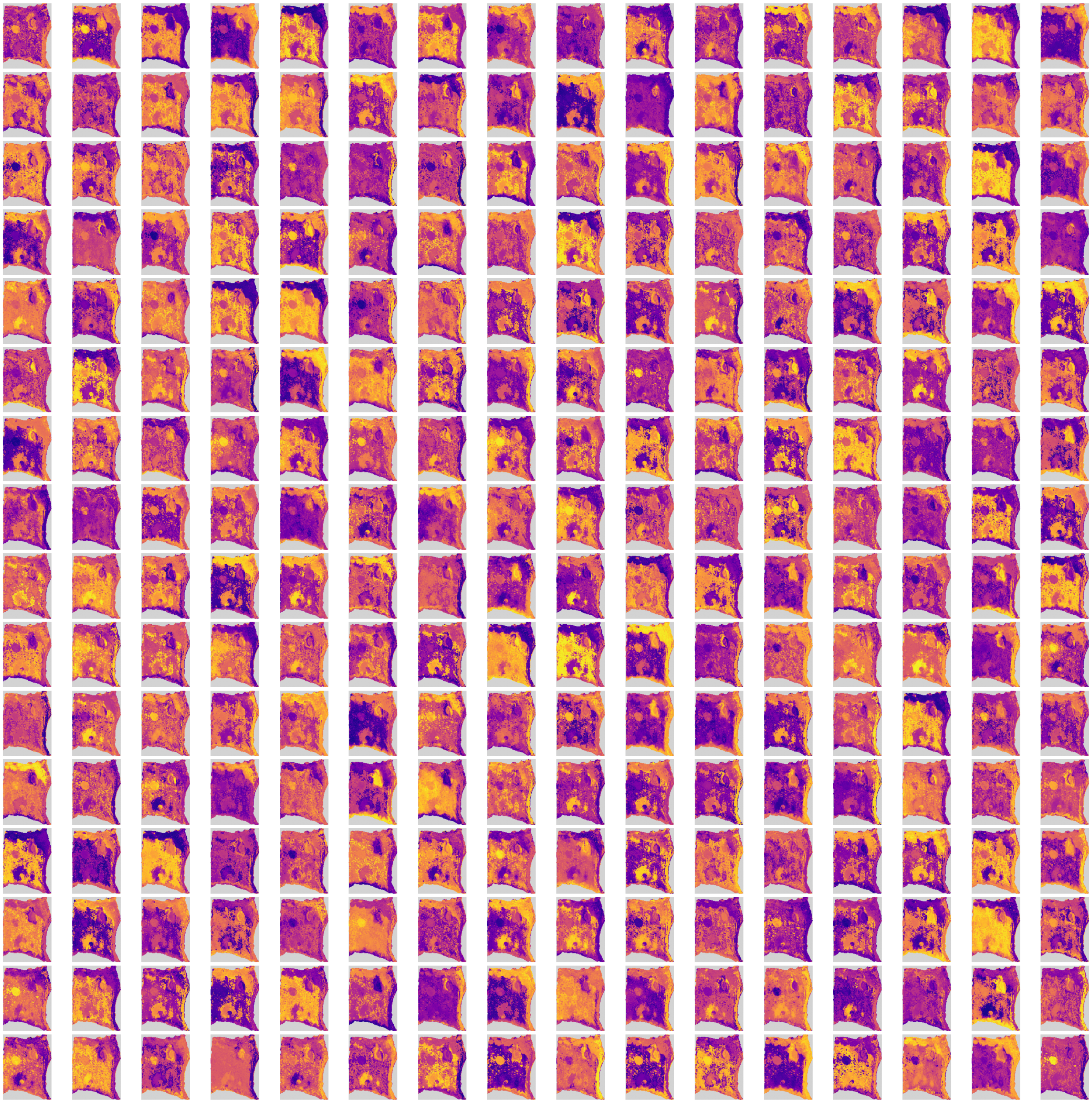

Figure 5: **256-dimensional encoded features of MSI data from the prostate cancer dataset.** The original high-dimensional data is compressed into 256 dimensions with minimal error, significantly enhancing the information on each dimension (see Supplementary Figure 1 for comparison). Furthermore, the distinctions between various zones within the prostate cancer tissue become discernible, thereby establishing a foundational basis for subsequent analyses.

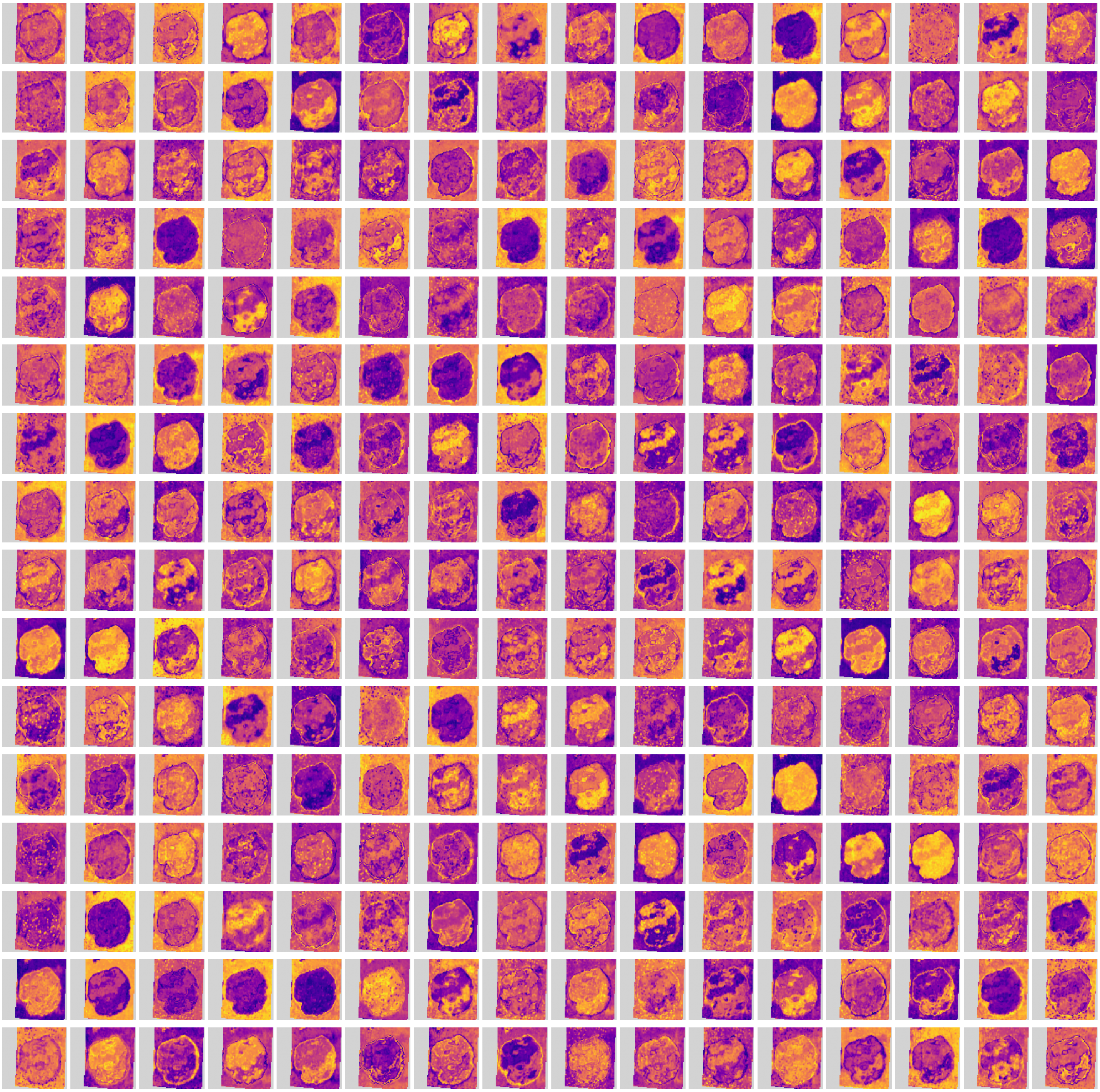

Figure 6: **Encoded features in training phase (Section 2)**. Low-dimensional encoded features efficiently capture molecular structures from original high-dimensional data.

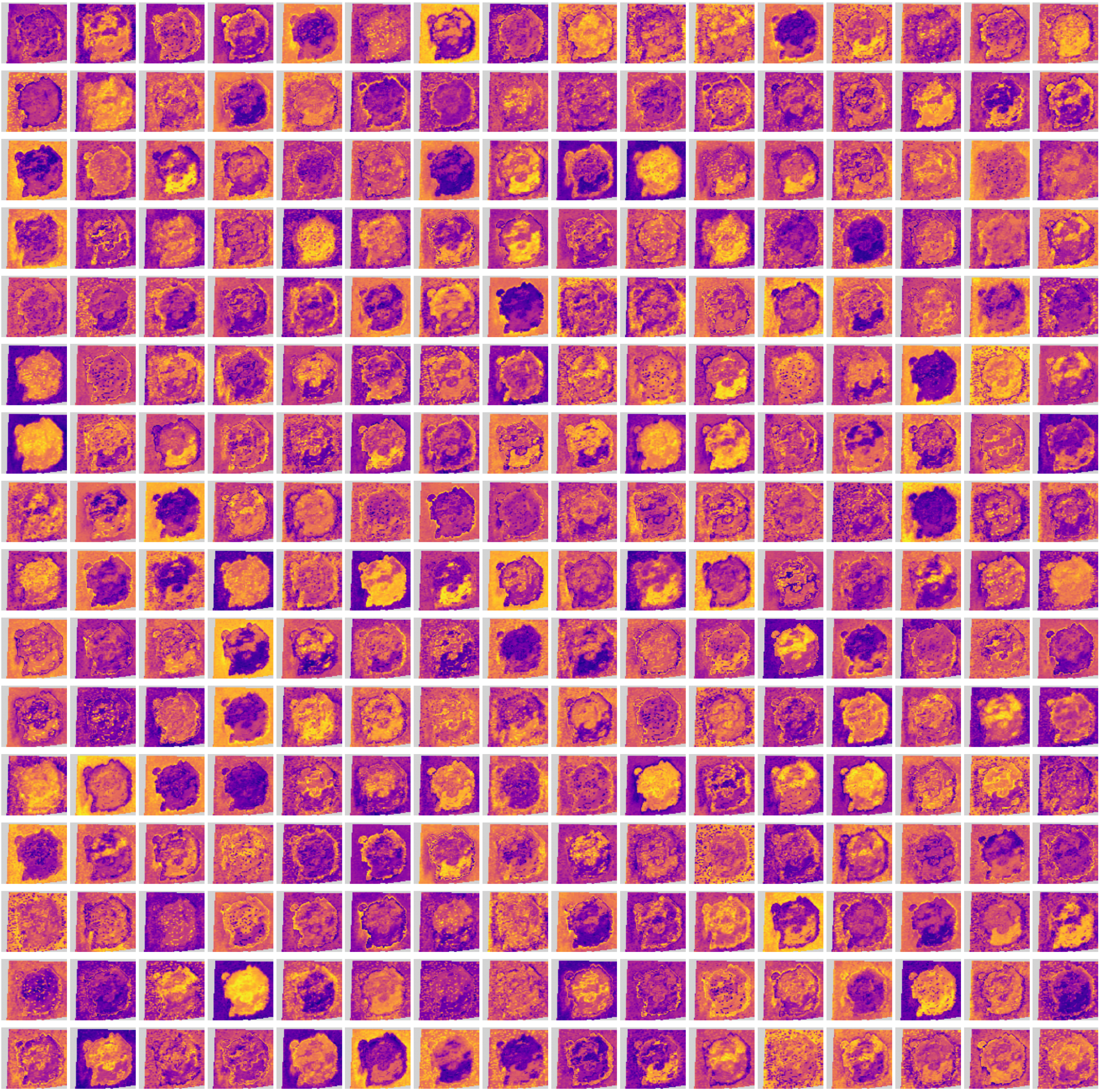

Figure 7: **Encoded features in testing phase (Section 4)**. Similar to the encoding features generated during the training process, the 256-dimensional features effectively integrate the nonlinear manifold within the raw data, yielding comparable outcomes. This similarity underscores the absence of significant overfitting in the model.

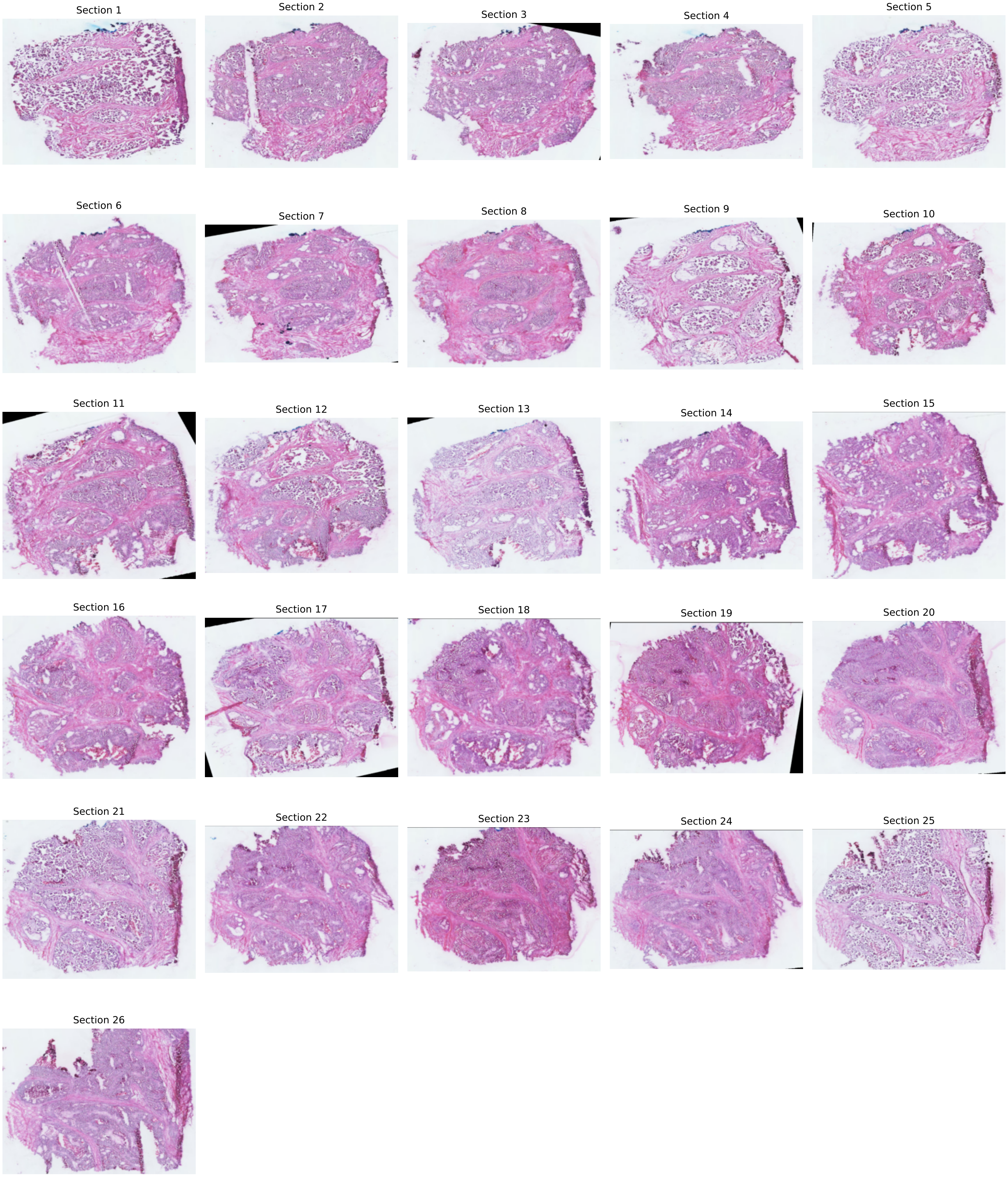

Figure 8: **H&E stained patterns of colorectal adenocarcinoma dataset (26 sections).**

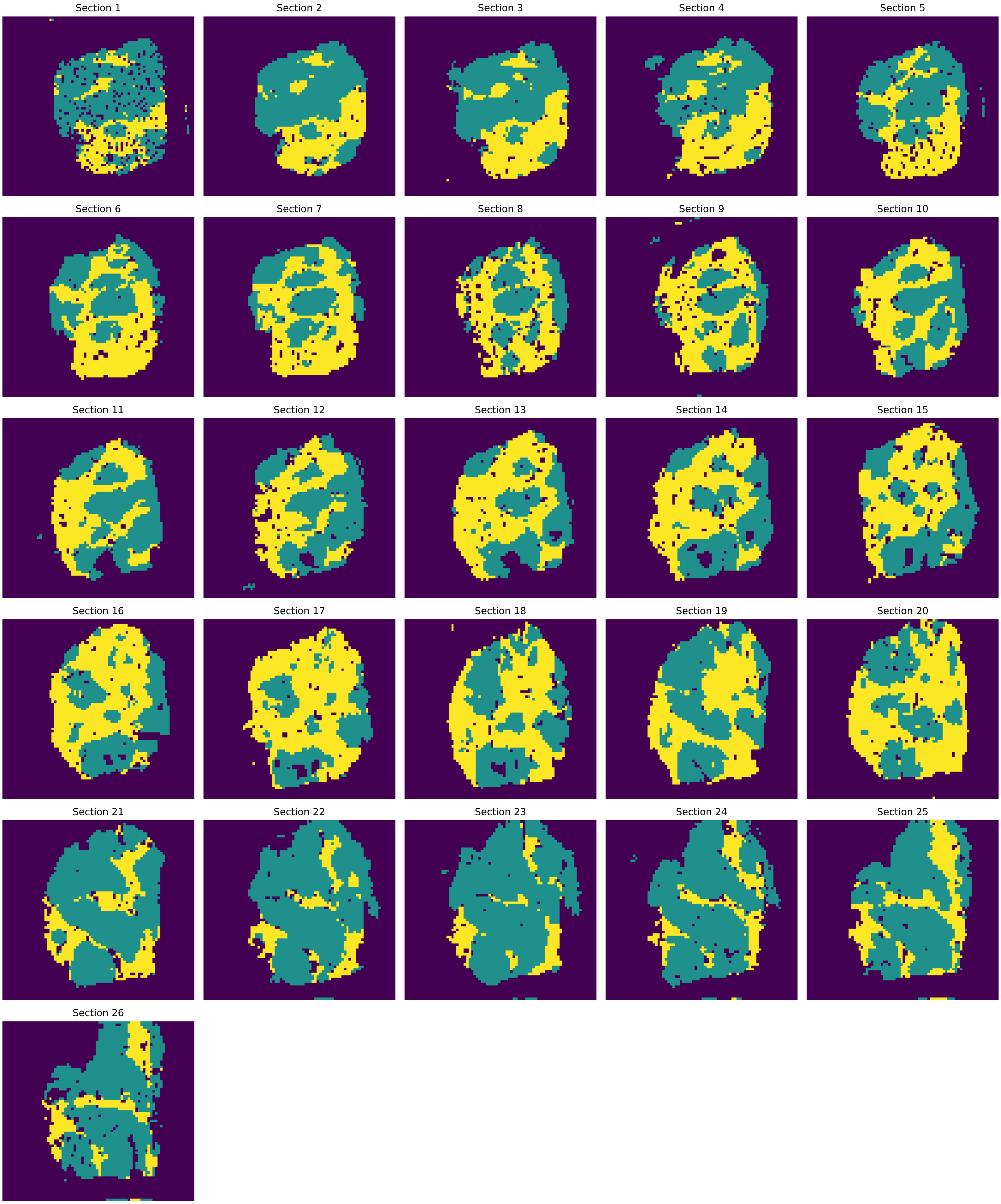

Figure 9: **Clustering results of colorectal adenocarcinoma dataset (Derived from Atnal)**. Tumor (blue) and connective tissue clusters (yellow) are selected from the clustered image which is derived from the encoded features using a Gaussian mixture model (k=5). These structures exhibit a notable resemblance to their corresponding regions in the H&E histology, as indicated in Supplementary Figure 8.

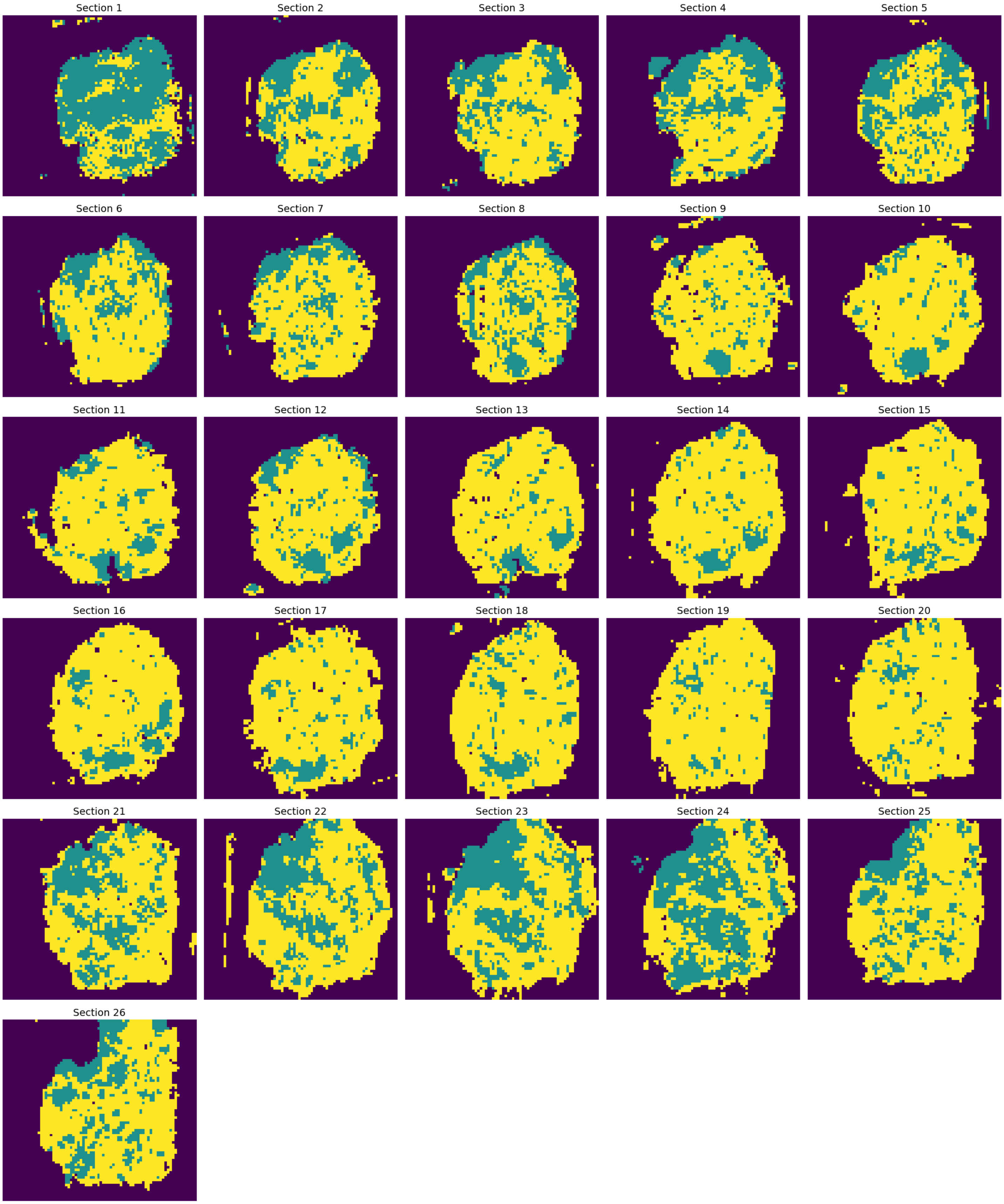

Figure 10: **Clustering results of colorectal adenocarcinoma dataset (Derived from PCA).**

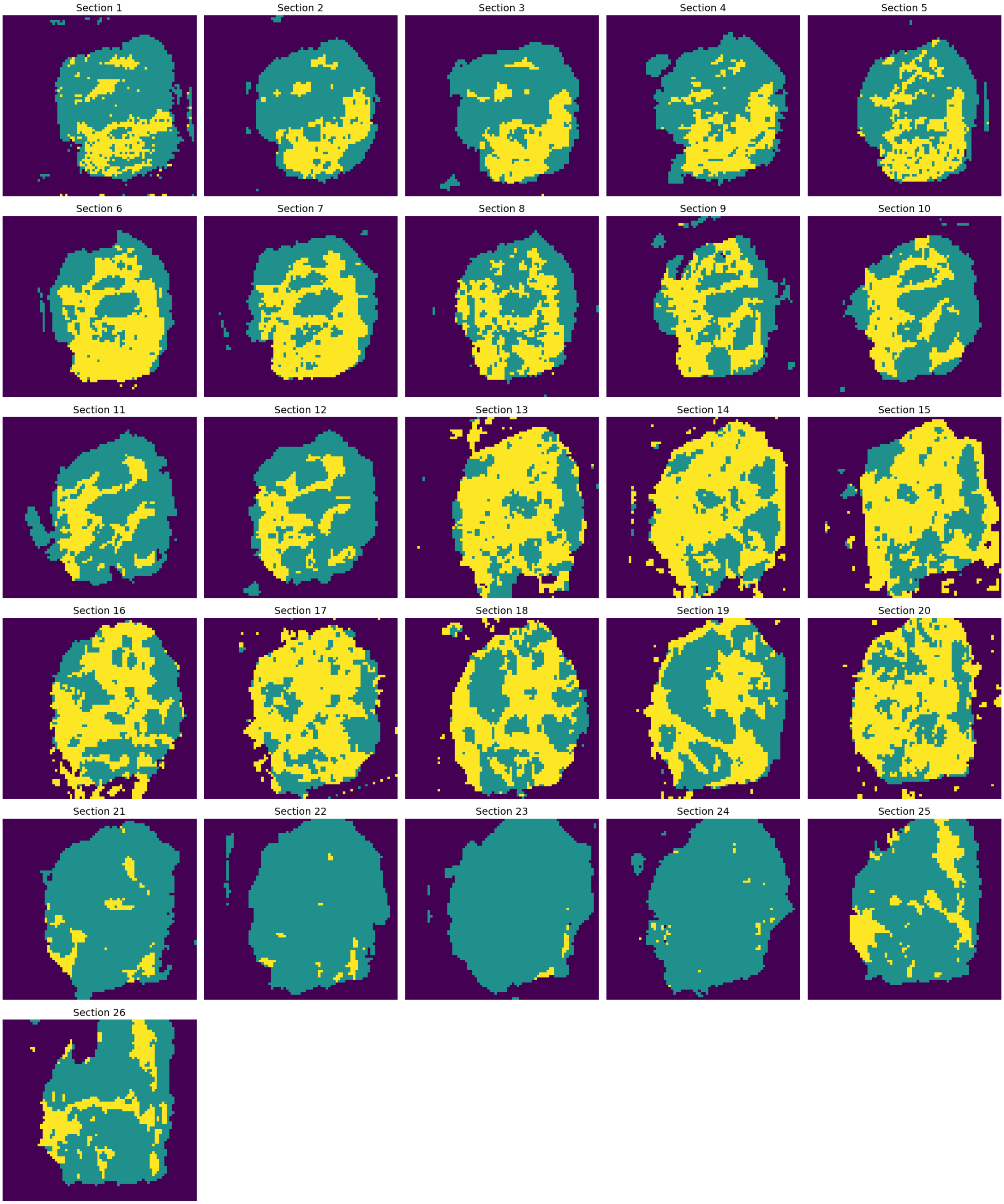

Figure 11: **Clustering results of colorectal adenocarcinoma dataset (Derived from t-SNE).**

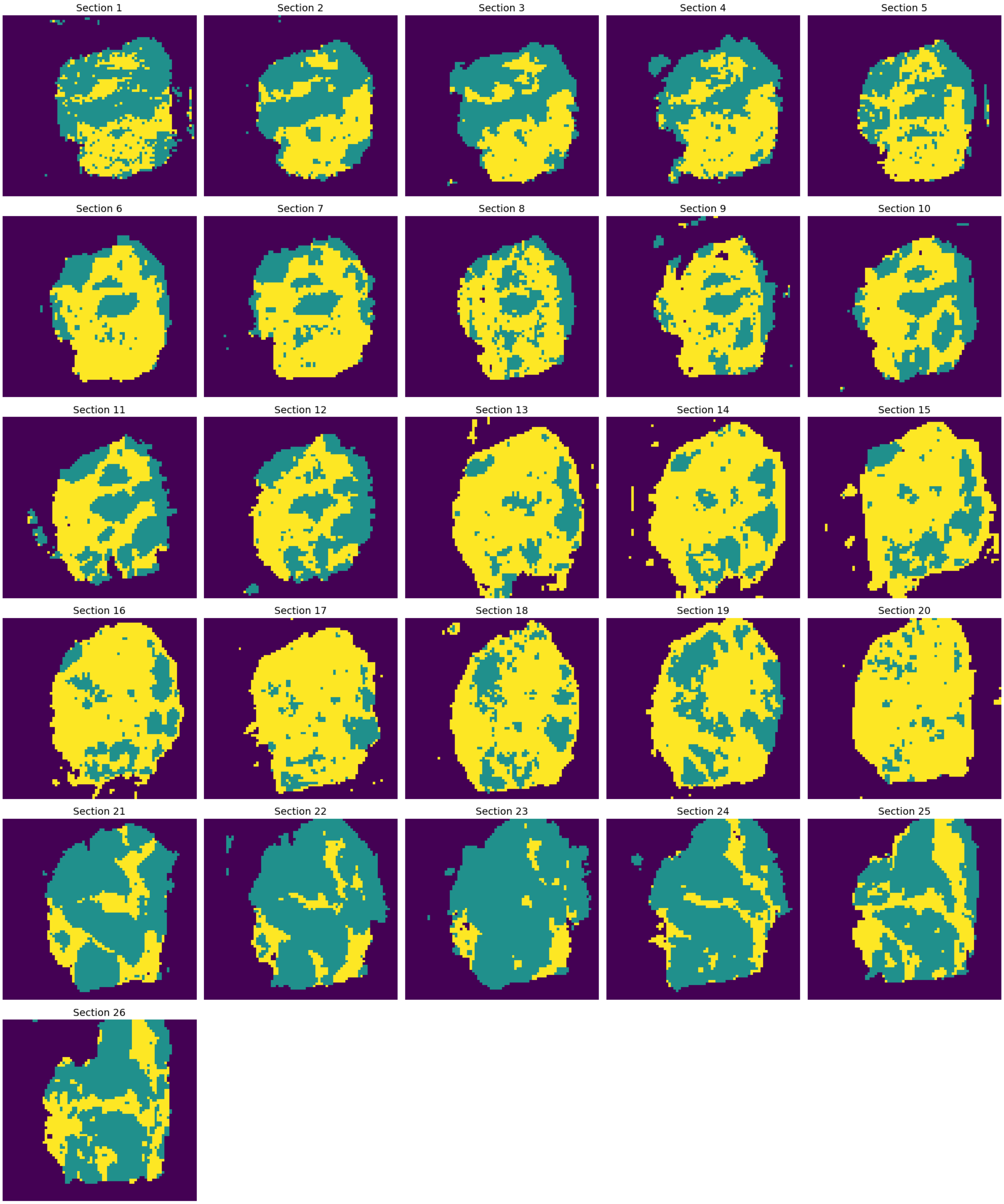

Figure 12: **Clustering results of colorectal adenocarcinoma dataset (Derived from GPLVM).**

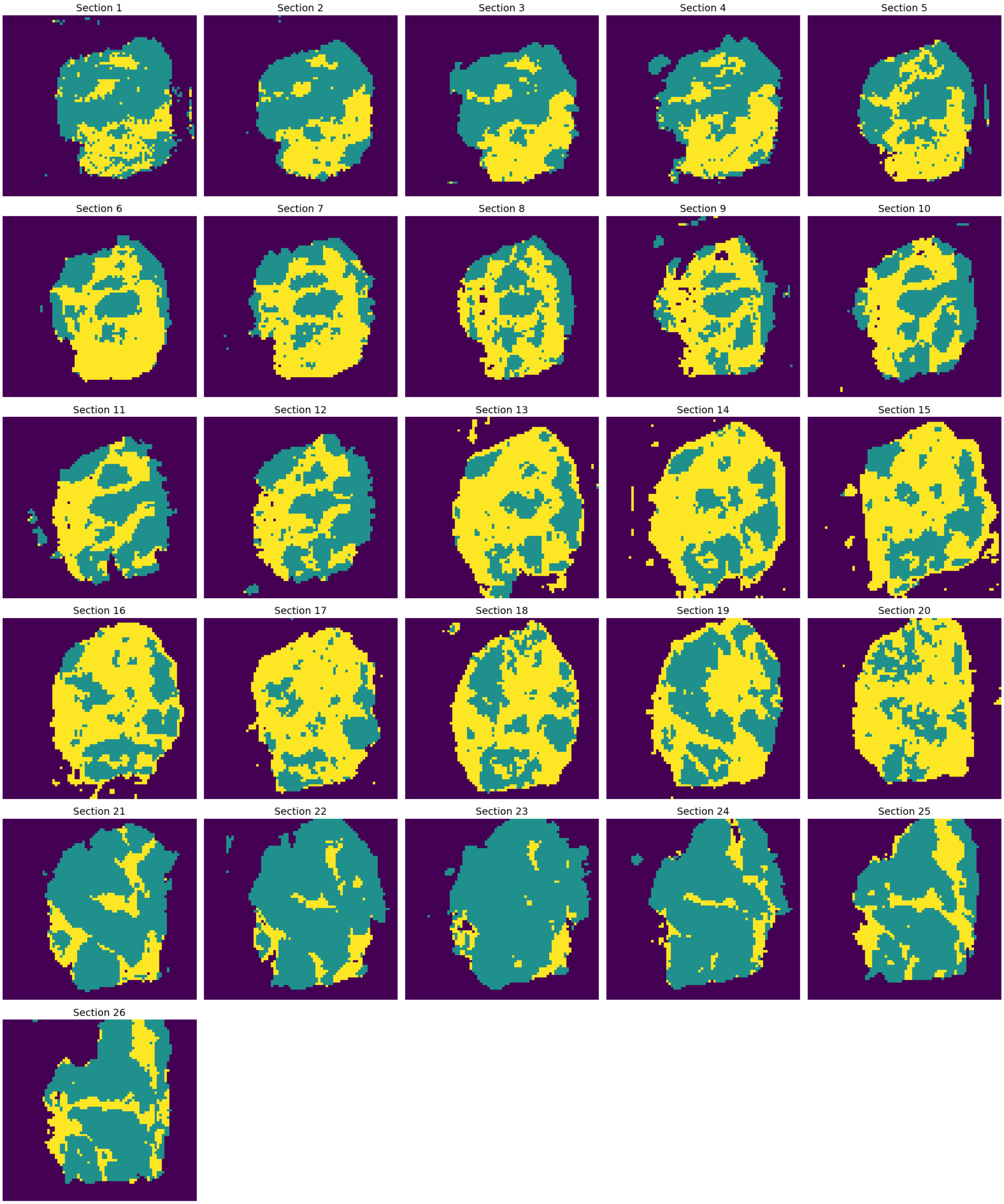

Figure 13: **Clustering results of colorectal adenocarcinoma dataset (Derived from MCML).**

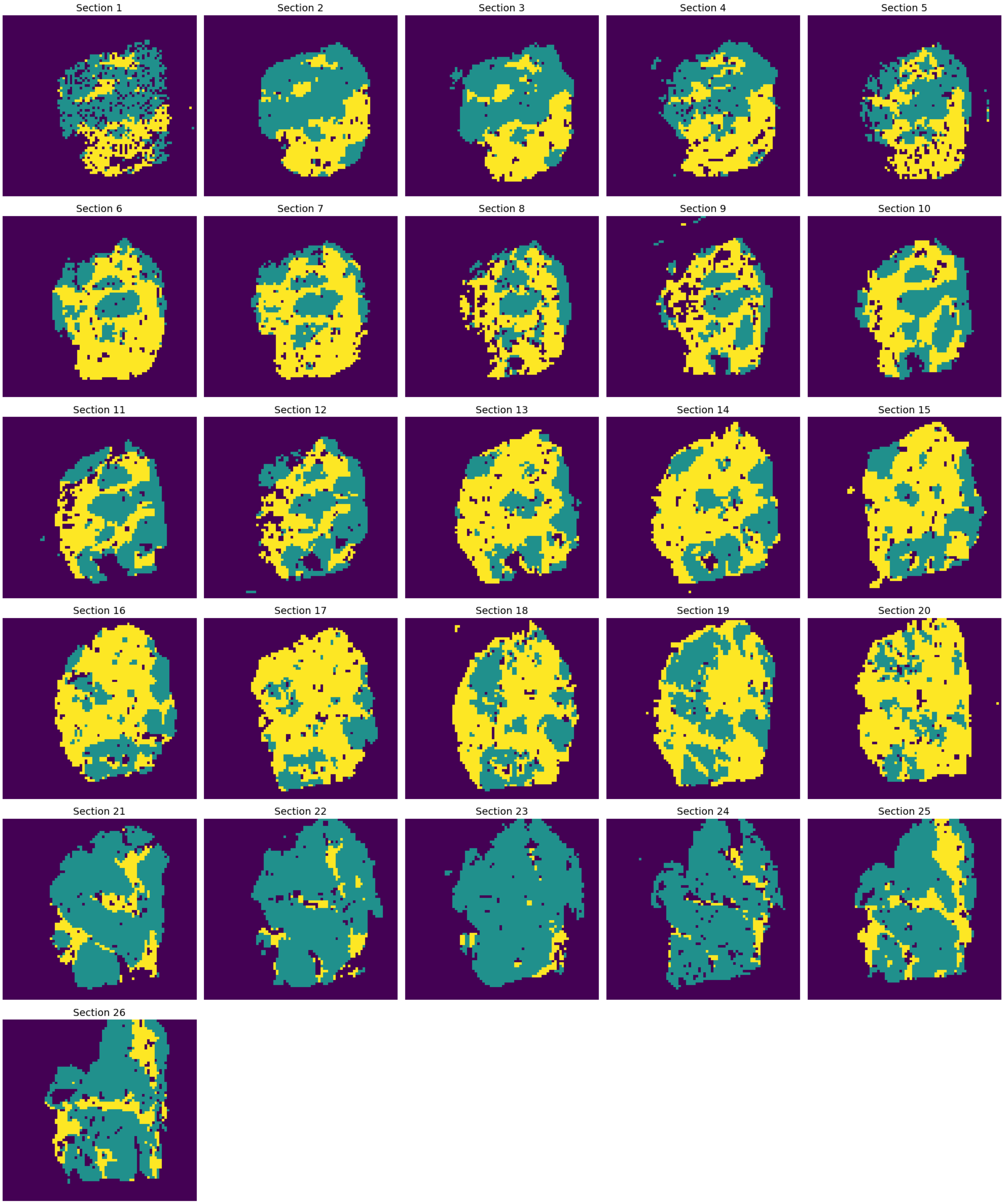

Figure 14: **Clustering results of colorectal adenocarcinoma dataset (Derived from msiPL).**

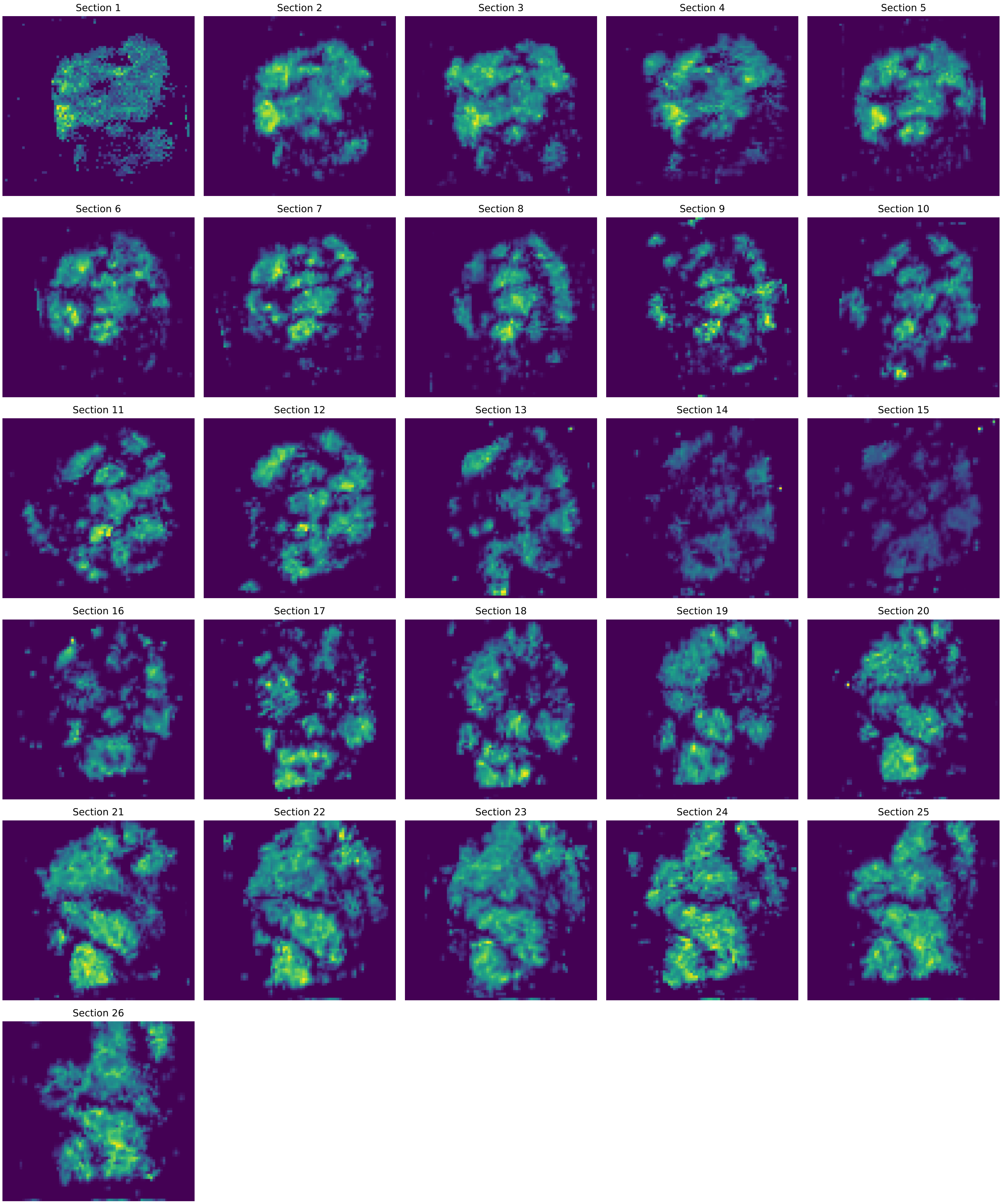

Figure 15: **The spatial distribution of the ion feature most highly correlated with the tumor cluster is found at m/z 720.49, with a Pearson correlation coefficient of 0.79.**
